# Biofortification—A Frontier Novel Approach to Enrich Micronutrients in Field Crops to Encounter the Nutritional Security

**DOI:** 10.3390/molecules27041340

**Published:** 2022-02-16

**Authors:** Salwinder Singh Dhaliwal, Vivek Sharma, Arvind Kumar Shukla, Vibha Verma, Manmeet Kaur, Yashbir Singh Shivay, Shahida Nisar, Ahmed Gaber, Marian Brestic, Viliam Barek, Milan Skalicky, Peter Ondrisik, Akbar Hossain

**Affiliations:** 1 Department of Soil Science, Punjab Agricultural University, Ludhiana 141004, India; ssdhaliwal@pau.edu (S.S.D.); sharmavivek@pau.edu (V.S.); vermavibha@pau.edu (V.V.); manmeetgill885@gmail.com (M.K.); saamina90-coasoil@pau.edu (S.N.); 2 ICA—Indian Institute of Soil Science, Bhopal 462038, India; arvindshukla2k3@yahoo.co.in; 3 Department of Agronomy, Indian Agricultural Research Institute (ICAR), New Delhi 110012, India; ysshivay@hotmail.com; 4 Department of Biology, College of Science, Taif University, P.O. Box 11099, Taif 21944, Saudi Arabia; a.gaber@tu.edu.sa; 5 Department of Plant Physiology, Slovak University of Agriculture, Tr. A. Hlinku 2, 949 01 Nitra, Slovakia; peter.ondrisik@uniag.sk; 6 Department of Botany and Plant Physiology, Faculty of Agrobiology, Food, and Natural Resources, Czech University of Life Sciences Prague, Kamycka 129, 165 00 Prague, Czech Republic; skalicky@af.czu.cz; 7 Department of Water Resources and Environmental Engineering, Faculty of Horticulture and Landscape Engineering, Slovak University of Agriculture, Nitra, Tr. A. Hlinku 2, 949 01 Nitra, Slovakia; viliam.barek@uniag.sk; 8 Department of Agronomy, Bangladesh Wheat and Maize Research Institute, Dinajpur 5200, Bangladesh

**Keywords:** mineral dense field crops, agronomic biofortification, green technology, transgenic/biotechnological approach, nanotechnology, gene modification

## Abstract

Globally, many developing countries are facing silent epidemics of nutritional deficiencies in human beings and animals. The lack of diversity in diet, i.e., cereal-based crops deficient in mineral nutrients is an additional threat to nutritional quality. The present review accounts for the significance of biofortification as a process to enhance the productivity of crops and also an agricultural solution to address the issues of nutritional security. In this endeavor, different innovative and specific biofortification approaches have been discussed for nutrient enrichment of field crops including cereals, pulses, oilseeds and fodder crops. The agronomic approach increases the micronutrient density in crops with soil and foliar application of fertilizers including amendments. The biofortification through conventional breeding approach includes the selection of efficient genotypes, practicing crossing of plants with desirable nutritional traits without sacrificing agricultural and economic productivity. However, the transgenic/biotechnological approach involves the synthesis of transgenes for micronutrient re-translocation between tissues to enhance their bioavailability. Soil microorganisms enhance nutrient content in the rhizosphere through diverse mechanisms such as synthesis, mobilization, transformations and siderophore production which accumulate more minerals in plants. Different sources of micronutrients viz. mineral solutions, chelates and nanoparticles play a pivotal role in the process of biofortification as it regulates the absorption rates and mechanisms in plants. Apart from the quality parameters, biofortification also improved the crop yield to alleviate hidden hunger thus proving to be a sustainable and cost-effective approach. Thus, this review article conveys a message for researchers about the adequate potential of biofortification to increase crop productivity and nourish the crop with additional nutrient content to provide food security and nutritional quality to humans and livestock.

## 1. Introduction

The global threat to nutritional security, due to the growing population, addresses the need to implement feasible and cost-effective strategies in the global food system. More than 2 billion people in the world are affected by the deficiency of key micronutrients such as Fe and Zn [1]. In addition, micronutrient deficiency in soil has been assessed across many parts of the world, thus limiting the nutrient uptake in plants and ultimately in humans. These micronutrients play a vital role in the functions of the human body; thus, insufficient intake have prominently negative biological effects. For livestock as well, a huge demand and supply gap exists due to insufficient and low-quality fodder production. Thus, nutritional security is a topic of grave concern from the health perspective of human beings and livestock [2]. The term nutritional security refers to the intake of food enriched with essential nutrients in an adequate amount. In developing countries, the largest proportion of the daily diet comprises mainly of staple crops [3]. This introduced the need for growing ultra-nourishing food to achieve nutritional security. Various ways such as medical supplementation, dietary diversification, and food fortification are available. Indeed, biofortification has been proposed as a promising tool to alleviate malnutrition as it allows selected nutrients to be added into the edible part of a particular crop for their ingestion by humans and animals.

To date, numerous field crops, which serve as staple food worldwide, has been a primary target of researchers for biofortification. Cereal crops including rice, wheat and maize, pulses, oilseed crops and fodder crops tend to accumulate nutrients supplied exogenously and thus have been biofortified. Literature studies so far demonstrated the vast research on biofortified crops loaded with essential micronutrients such as Zn, Fe, I, Cu etc. [4]. In 2017, PAU released Zn-efficient PBW 1Zn variety, which possesses high Fe (40.0 mg L^−1^) and Zn (40.6 mg L^−1^) concentrations. Numerous biofortified crops including maize, sweet potatoes, orange, cassava, squash, sorghum, Fe-enriched beans, lentils, Zn-enriched rice, lentils, wheat, sorghum and cowpeas have been released [5]. Cakmak and Kutman compiled a review on agronomic biofortification of cereals enriched with Zn [6]. In another report, Fe fortified staple crops to overcome its deficiency has been studied by Connorton and Balk [7]. Another report elaborated the studies on agronomic iodine biofortification of leafy vegetables grown in Vertisols, Oxisols and Alfisols [8]. Zheng et al. focused on the carotenoids, lipophilic isoprenoids, fortified food through various approaches [9]. Meanwhile, the worldwide research to enrich the Se content in dietary crops through various strategies was compiled by Saeid et al. [10]. 

Various avenues currently in practice to achieve nutritional security are genetic engineering, conventional breeding and agronomic biofortification. The conventional approach refers to selecting existing varieties of high-yielding crops and cross-breeds with variety possessing higher nutrient content to produce staple crops with desirable nutrient and agronomic traits. Genetic biofortification can be attained through specific genetic manipulation to enrich micronutrient concentration in edible plant parts. Agronomic biofortification refers to the application of micronutrient fertilizers through soil application, foliar feeding or seed treatment to enrich the edible part of field crops with micronutrients [10]. Numerous management approaches such as the selection of suitable crops and cropping systems, application of lime, elemental sulfur and other organic amendments have also been practiced to improve the soil conditions that enhance the micronutrient bio-availability for plant uptake. 

The current scenario on global food demonstrated the insufficient quantity of multiple micronutrients in dietary crops; thus, the fortification of crops with a single nutrient is not enough to meet the nutritional security, especially where the diet is based on a limited range of food. Advanced studies were carried out for the application of mixed nutrients in crops. Recently, Górniak et al. [11] reported the studies in the field of biofortification of edible plants with selenium and iodine. Another factor that contributes towards the nutrient intake in the human body is whether the food consumed supplies micronutrient form in bioavailable form. This issue is more significant in the case of Fe and Zn where phytate acts as an inhibitor in the absorption of mentioned nutrients. Additionally, foliar feeding of several metal cations (Zn^2+^, Fe^2+^ and Cu^2+^) reduced the mineral content of other nutrients. Thus, synergistic and antagonistic relations of these nutrients are to be kept in view while progressing towards nutritional security. 

Nowadays, much attention has been paid to high-impact technologies such as nanotechnology that pave the way towards boosting agricultural production. Given the unique properties of nanomaterials (NMs) over their bulk counterparts such as higher surface-to-volume ratio and sorption capacity, targeted delivery and controlled-release kinetics, nano-fertilizers have been proved efficient to satisfy the imperative nutrient requirements of plants. Zulfiqar et al. [12] highlighted the importance of ENMs as an alternate solution to conventional chemical fertilizers and their positive role through slow-release fertilizers. Torre-Roche et al. [13] reported the impact of seed treatment with engineered nanomaterials (ENMs) on the quantitative and qualitative traits of plants. Various types of NMs such as nanotubes, metal NPs (Al, Cu, Ag, Au), metal oxide NPs (Fe_3_O_4_, ZnO, Ce_2_O_3_, TiO_2_) have been employed in agriculture. Another environmentally friendly approach for nutrient enrichment of plants is microbe-mediated biofortification due to the association between microbes and nutrients. Literatures studies have reported the positive influence of endophytic microbes including *Bacillus*, *Klebsiella*, *Acinetobacter*, *Rhizophagus intraradices*, etc. in improving the status of Zn and Se in *Triticum aestivum*. Plant growth-promoting rhizobacteria have also been employed in the form of biofertilizers or bio-inoculants for improved plant growth, directly or indirectly [14].

The present review summarizes the research progress on micronutrient biofortification of staple crops for nutritional security, followed by the different approaches and factors affecting the nutrient status including source and mode of mineral fertilization. We also propose to harness the full potential of biofortification through recent strategies such as nanotechnology or green technology.

## 2. Global Status of Micronutrients in Soil

Micronutrients deficiency in soil has been assessed through various analytical techniques across many parts of the world. The deficiencies primarily occur due to the excessive use of fertilizers that high-yielding crop varieties demand, along with the lack of micronutrient supplementation. For micronutrient deficiencies in important parts of world soil, maximum deficiency of Zn and B was observed to be 49% and 31%, respectively [15]. Other mineral deficiencies were found to be 15%, 14%, 10% and 3% for Mo, Cu, Mn and Fe, respectively. Boron plays a crucial role in mechanical resilience, as well as strengthening the cell membrane, and is present in BO_3_^−3^ form in soils. Indicators for low B availability in soil have been recorded in a few regions in almost every country, particularly in Nepal, India, the Philippines and Thailand. The well-known role of Cu as a catalyst involves protein and vitamin A synthesis as well as enzyme activation in several plant-growth processes. Copper toxicity has been widely observed in the soil from Brazil, the Philippines, Tonga and Italy. 

Iron is primarily known as a component for hemoglobin and many other enzymes involved in nitrogen fixation, energy transfer and lignin synthesis. Inadequacy in Fe bio-available form in soil has been observed in Mexico, Malta and Turkey and a few localities of several other countries. Another micronutrient, Mo, acts as a structural component of nitrogenase, which is a key enzyme in nitrogen fixation in rhizobium legume symbiosis. Deficiency of Mo occurs frequently in areas with acidic soils, as observed in Africa (Zambia, Sierra Leone, Ghana, Nigeria), besides Nepal, Brazil and New Zealand. Micronutrient Mn is known primarily for its role in facilitating the photolysis of water molecules during photosynthesis and nitrogen metabolism, as well as increasing the availability of P and Ca in plants, although excessive Mn level decreases the uptake of Ca in plants [16]. Alkaline soils are found to be Mn deficient, whereas acidic soils are found to possess sufficient Mn content, thus the pH of soil affects the availability of Mn. As observed in Syria, India, Egypt, Pakistan, Italy and Lebanon with alkaline soils, high Mn deficiency exists. Zn deficiency has been observed frequently worldwide; however, it is more prevalent in Iraq, Pakistan, India, Turkey, Syria, Mexico, Italy, Lebanon, Nepal, Tanzania and Thailand. 

In most of the Indian soil, micronutrients are present in sufficient amounts; however, their bio-availability for plant uptake is low; thus, soils in several Indian localities are inadequate. Boron deficiencies exist in an irregular pattern in Indian soils, ranging from 68% deficiency in red soils of Bihar to 2% in alluvial soils of Gujarat. Maximum B deficiency (54–86%) was observed in Alfisol soils of West Bengal and Assam, due to high rainfall that leads to a decrease in water-soluble B. Indo-Gangetic plains with saline soil exhibited a higher concentration of B, whereas the moderate level of B was recorded in Rajasthan and Madhya Pradesh. Copper deficient soil was observed in Kerala, Himalayan Tarai zone, Bihar, Uttar Pradesh and north Madhya Pradesh [17]. 

Despite the higher abundance of iron in the earth’s crust, its plant-available concentrations are low in the alkali soils of Indo-Gangetic regions. Soil analysis reports demonstrated that approximately 12% of Indian soils are Fe deficient. Manganese-deficient soil is rarely observed in India as only 1–5% of surface soil samples were found to be Mn deficient. Manganese deficiency was primarily observed in Haryana, Bihar, Punjab as well and Madhya Pradesh. Additionally, excess application of lime in red lateritic soils of Orissa resulted in Mn deficiency [18]. Molybdenum deficiency has been observed 11% in total Indian soil including hill soils of Andhra Pradesh, Konkan and Malabar regions and north and northeastern Himalaya regions [18,19]. On the contrary, calcareous alkaline soils of the Punjab region possess high available Mo contents and thus potential toxicity can be observed in crops. As observed, wheat straw as forage possessed high Mo contents and was thus found to be toxic to cattle. Out of 65,000 soil samples of India, 51.2% samples were found to be Zn deficient; thus, Indian soils are the most deficient in Zn in the world, with a widespread deficiency in Indo-Gangetic plains. The decline in Zn deficiency has been observed in the states such as Haryana, Punjab, Uttar Pradesh, Bihar, Andhra Pradesh and Madhya Pradesh.

## 3. Global Status of Micronutrients Malnutrition

**Figure 1:**
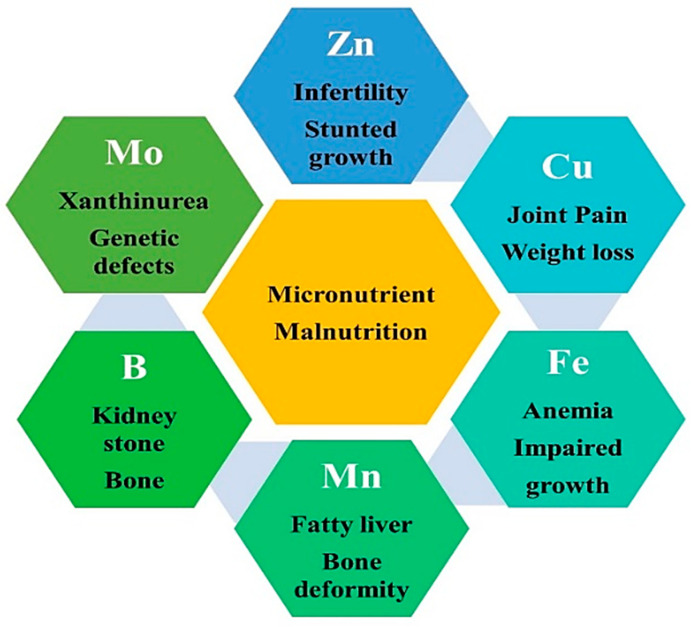
Effects of micronutrient malnutrition on human health.

The invisible form of micronutrient malnutrition also referred to as ‘Hidden Hunger’ has affected one in three people globally. The insidious effects are observed in particular areas where a lack of dietary diversification exists, especially in developing countries. Vitamin A, Fe, I, Zn and folate deficiency are of major concern for global human health and the effects of various micronutrients on human health have been shown in Figure 1. 

The evolution of the concept of malnutrition originated in the early 20th century. During the 19th century, the importance of trace elements such as Fe, Zn and I from health perspectives was well-established. Further, the concept of multiple micronutrient nutrition and their dietary requirement was addressed in the 1940s. After World War II, multiple micronutrient deficiencies were observed at a large scale in developing countries. To combat micronutrient malnutrition, the Rome-based World Food Conference called for action in 1974 [20]. However, due to less activity on the global level, intense concern originated from the scientifically sound epidemiologic surveys data between 1975 and 1985 for micronutrient deficiencies. 

Professional alliances named International Nutritional Anemia Consultative Group, International Vitamin A Consultative Group and the International Council for Control of Iodine Deficiency Disorders were formed, including some international and bilateral donor agencies WHO, FAO and the World Food Programme, national program managers and the private sector. In 1985, a 10-year UN agency plan was launched to eliminate I, Fe and vitamin A deficiencies. The World Summit for Children in 1990, held by the UN, with contributions from various regulatory bodies such as UNICEF, WHO, the World Bank, FAO, etc. was a significant event to address and achieve nutritional security by 2000. Afterwards, within the decade, three summits were held with the goals to eliminate deficiencies in essential nutrients such as iron, iodine and vitamin A. In 1991, a meeting, “Ending Hidden Hunger”, was held in Montreal by the WHO-UNICEF joint initiative. Another micronutrient expert group, the IZiNCG, which aimed to reduce the Zn deficiency worldwide, was founded in 2000. In 2001, the Millennium Development Goals resolution to combat the world’s chief health and poverty issues by 2015 was adopted by the General Assembly of the UN. Since 2003, the Consultative Group on International Agricultural Research initiated the Harvest Plus program, which has made remarkable progress in the area of biofortification to fight against micronutrient deficiency. In April 2010, the “Scaling up nutrition” meeting was hosted by USAID and World Bank between the governments of Canada and Japan, to mobilize investment in nutrition interventions. Thus, the current scenario showed the involvement of various global normative and regulatory agencies, government institutions, NGOs and private sectors to eradicate micronutrient deficiency in the human body.

Three major approaches to achieve nutritional security are dietary diversification, medical supplementation and biofortification. The first strategy deals with the increase in the diversity of food intake. Starchy staple crops are dominating diets worldwide, with special reference to developing countries. Thus, the exclusion of fruits and vegetables, pulses and animal proteins makes the diet nutritionally deficient. However, practicing ‘dietary diversification’ is not feasible in these countries, especially among low-income populations. The second approach refers to the artificial addition of nutrients, either by supplementation or by fortification of basic food products, e.g., iodized salt. The common examples include vitamin A added to cooking oil and iron- and folate-fortified flour. However, various factors that limit the implementation of these approaches are infrastructure, robust distribution, purchasing power, storage and consumer compliance. Thus, the biofortification approach is most sustainable and cost-effective under ideal situations to ascertain the nutritional security goal. 

Zinc deficiency has affected more than one-third of the world’s population. Its adequate intake is mandatory to perform various physiological functions in the human body, such as immunity fertility, normal vision and wound healing, and also for proper growth in childhood to inhibit the infections such as malaria, pneumonia and diarrhea [21]. Iron deficiency is another worldwide problem associated with nutritional disorders in the diet. Iron is known for its contribution to hemoglobin and the formation of red blood cells. A diet lacking in Fe in the human body leads to anemia, impaired physical and mental development that further enhances the susceptibility to malaria, HIV/AIDS and tuberculosis [21]. Iodine is known for its function in the synthesis of thyroid hormones and its deficiency leads to goiter problems in the human population, also known as iodine deficiency disorders. Its deficiency is a widespread malnutrition problem; thus, its administration is necessary. Folates, a group of water-soluble B vitamins, have a predominant role in primary metabolism; thus, detrimental physiological effects occur upon folate deficiency. Enzymes utilize folate in thymidylate, purine synthesis and pantothenate (vitB5) formation. In plants, folates have a vital role, being involved in photorespiration, chlorophyll, plastoquinone, tocopherol, pectin and lignin synthesis. Besides the common role of selenoproteins in the antioxidant defense system, Se plays an essential role in the immune system, cardiovascular diseases, impaired fertility, inflammatory disorders diabetes, and many types of cancer. Its deficiency is associated with Keshan disease failure and Kashin-Beck disease [10]. Thus, the concept of nutritional security has emerged as a major concern where there is a high reliance on staple crops. 

### 3.1. Malnutrition in Young Infants and Adult Humans

Apart from devasting health consequences, micronutrient deficiencies (such as Fe, Zn, I and vitamin A deficiency) are projected to cost around 0.8–2.5 per cent of the gross domestic product [22]. The Global Burden of Disease Study 2016 report projected iron-deficiency anemia among the top 10 causes of disability-adjusted life years for women. Iron deficiency-induced anemia is prevalent globally, affecting over 30% of the world’s population, including 33% of non-pregnant women, 40% of pregnant women, and 42% of children worldwide [23]. According to WHO estimation, 20% of maternal deaths are attributed to anemia alone. In contrast to anemia, which is more common in women, Zn deficiency can be prevalent in men, women and children. Globally, the relative statistics significantly vary in rates of Zn deficiency. Among high-income regions, including North America, Europe, Oceania and Central Asia, Zn deficiency is observed below 5–10% of the total population, whereas across Sub-Saharan Africa and South Asia it varies between 15 and 50%, with the highest prevalence 54% in the Democratic Republic of Congo. Globally, 250 million preschool children are estimated to be vitamin A deficient and 250,000 to 500,000 vitamin A-deficient children become blind every year, half of them dying within 12 months of losing their sight. Across North Africa, the Middle East and Central/East Asia, between 20 and 25% of pregnant women were vitamin-A deficient. Most countries across Sub-Saharan Africa and South Asia had slightly lower rates between 15% and 20%. Iodine deficiency is the major cause of brain damage in children that occurs during fetal development and in the first few years of a child’s life. Globally, iodine deficiency has affected 30% of the world’s population. Other elements deficiencies, such as Ca, vitamin D and folates, are observed during pregnancy, leading to health complications for both the mother and growing baby.

In India, around 0.5% of total deaths in 2016 were attributed to nutritional deficiencies; according to the National Family Health Survey (NFHS4), carried out by the Ministry of Health and Family Welfare, the anemia rates observed among different groups were as follows: children (6–59 months), women (15–49 years), pregnant women (15–49 years) and men (15–49 years), at a rate of 58.6, 53.1, 50.4 and 22.7 per cent, respectively (micronutrient status of the Indian population). Iodine deficiency disease (IDD), though, declined in recent years through iodized salt, yet it exists particularly across the Sub-Himalayan regions. Among severe vitamin-deficiency diseases, some of them, including pellagra (niacin deficiency), beri (thiamine-vitamin B1 deficiency) and scurvy (vitamin C deficiency), have disappeared. However, milder clinical manifestations and biochemical (sub-clinical) evidence reflected the existence of blindness and rickets due to vitamin A and vitamin D deficiency, respectively. Among South Asian countries, 62% of preschool children were found to be vitamin A deficient, leading to an annual 330,000 child deaths [24]. The copper level survey in India reported 29–34 per cent deficiency among pregnant women and the adult tribal population. Zinc deficiency was found to be highly prevalent among pregnant women (64.6%), followed by adults (49.4%) and children aged 6–60 months (43.8%) (micronutrient status of the Indian population).

### 3.2. Malnutrition in Animals

Livestock is a unanimous part of human life that plays important agricultural and socio-economic roles. The total world population of cattle is estimated to be 987.5 million heads [25]. India ranks first in cattle population, as 30.70% of the total population belongs to India, followed by Brazil and the US. However, the larger portion of land is modeled for the production of food and cash crops and an insufficient portion of arable land is available for fodder production. Indian livestock requires 883.95 Mt of green fodder and 583.66 Mt dry matter of fodder; however, present production is 664.73 Mt of green fodder and 355.93 Mt of dry fodder (million tonnes) [26]. Thus, to cover the demand–supply gap, high-quality fodder production enriched with nutrients is mandatory. Like humans, animals too require micronutrients for optimum growth and productivity. However, these microelements rarely present in sufficient amounts in the food vehicle, i.e., soil, fodder crops, animals, and ultimately in the end-users, i.e., humans. Thus, supplemental minerals are habitually added to animal feed as the nutrient need is hardly ever met by plant foods alone. Anti-nutrients such as phytic acid and tannins suppress the bioavailability of micronutrients by inhibiting their absorption in the animal gut. 

Since green fodders are a rich source of Fe, its deficiency rarely occurs in farm animals. The consequences of Zn deficiency in animals results in reduced growth, poor appetite, reproductive problems and swollen feet with open scaly lesions and impaired reproduction [27]. Symptoms of Zn deficiency appear in the form of hair loss, dermatitis, parakeratosis, deformed limbs, stiff joints, etc. On the contrary, Cu deficiency symptoms have appeared in the form of hair around the eyes of cattle, uncoordinated gait in lambs, crimpled wool in sheep and heart failure leading to death in cattle [27]. Boron plays a crucial role in improving the immune system and in the functioning of hormones, steroids, vitamins and minerals animal body system. To date, no specific symptoms of B deficiency have been observed in animals. Mo is associated with several enzymes in animals other than those mentioned in animals. In grazing animals and ruminants, excess Mo intake results in Cu deficiency leading to molybdenosis in animals. In the digestive tract of ruminants, the formation of thiomolybdates, a compound containing Mo and S, prevents the absorption of Cu, leading to its deficiency, which can be rectified by sulfate supplementation [28]. The consumption of micronutrient, deficient animal food source will eventually affect the nutritional status of the human body. Thus, increasing consumption of animal-source foods using improved fodders contributes to the economic and health benefits of human beings.

## 4. Field Crops as a Rich Source of Minerals

To eradicate malnutrition, the dietary pattern must incorporate the minimum required amount of essential nutrients including carbohydrates, fat, proteins, vitamins and minerals [29]. To meet this requirement, various types of crops are grown globally, including cereals, pulses, oilseeds and fodder crops. Due to the lack of a diverse diet in developing countries, the staple crops must contain essential nutrients in an adequate diet. Although more than 50,000 edible plants exist globally, 90% of the food intake is provided by 15 of them. Among them, rice, maize (corn) and wheat contribute to two-thirds of world staple crops intake. Apart from them, millet and sorghum, cassava, potatoes, yams, taro, dairy products and animal products such as meat, fish are major dietary intakes. The principal diet in low-income developing countries constitutes cereals as staple food, including wheat, while rice, millet, corn and sorghum. 

Rice is a major food staple worldwide, particularly in Asia, Latin America, and parts of Africa, whereas maize is a native crop of Central America. In South Asia, the particularly vegetarian community largely reliance on legumes. Wheat is the chief dietary crop in North Africa and west and central Asia regions, whereas in Sub-Saharan Africa it is sorghum and millet. Conventionally, food staples depended on the geographical features of the region. However, with improved agricultural aspects, transportation and food storage, global variation in food staples has been observed. For example, quinoa, a grain-like plant, was traditionally grown in the Andes Mountains of South America, yet now consumed outside of Latin America.

### 4.1. Cereals Crops

Apart from global food dependency, cereal grains can absorb more mineral nutrients in their edible parts. Thus, the scientific community emphasizes improving the profile of cereal *grains* concerning micronutrients. Among cereal grains, rice is consumed by more than half of the world population and contributes to more than 42% of calorie intake [1]. About 90% of global rice consumption is in Asia and is the fastest-growing staple food in Africa and Latin America. The nutritional composition of rice consists of several vitamins (such as vitamin B1, B2, B3, B6), diverse phyto-molecules and various minerals such as Na, Zn, Fe, Cu, K, Mg and P. The second staple food after the rice is wheat, in South Asia, Turkey and China, and contributes to more than 70% of daily calorie intake. Thus, its nutritional value is of utmost importance to meet nutritional security. Wheat grain typically consists of the testa, the aleuronic layer, the embryo and the endosperm. The embryo is the living part of the grain and is very rich in nutrients. The aleuronic layer consists of most of the nutrients (e.g., protein, minerals and vitamins), whilst the endosperm is concentrated mainly with starch [30]. Its diverse functionality enhances its importance as its seeds can be grounded into semolina, flour, etc., which are the basic ingredients of pasta, bread and bakery products. Corn, also termed as maize, is the world’s leading staple crop along with wheat and rice. Its popularity is largely due to its use as a food source for both humans and animals. It can be consumed in many ways, such as boiled, roasted, fried, ground and fermented, for use in gruel, bread, cakes, porridges and alcoholic beverages. In industries, its use is in sweeteners, oils, food thickeners and non-consumables. Its nutritional content consists of 72% starch, 10% protein and 4% lipid. Maize contains numerous important vitamins except for vitamin B-12. Eighty percent of the total mineral content of maize resides in germ and less than 1% in the endosperm. The most prevalent minerals found in maize are K, Mg, Zn and P (in the form of phytate). Sulfur is present in the form of methionine and cystine. Ca and Fe are found in negligible amounts and other trace minerals include Mn, Se, Cu and I [31].

### 4.2. Pulses Crops

Pulses are also termed as a superfood due to their intense nutritional composition providing multiple health benefits. Their acknowledgment to achieve nutritional security can be recognized as they were declared the theme of the year 2016 by FAO (Food and Agricultural Organization of the United Nations). After cereals, they rank as the second major crop production in the agricultural sector worldwide, with major consumption (over 70%) of lentils, peas, beans and chickpeas worldwide [32]. Subsequently, their importance is highlighted for socio-economic impact attributed to their inter-cropping systems, with cereals contributing to the sustainability of crops systems, low fertilizing and water requirements, high resistance to diseases, prolonged storage time and adaptability to extreme conditions [33]. Together, these factors hold technological/functional properties in wide industrial applicability for functional food development. 

Globally, six pulses, namely, chickpea (*Cicer arietinum*), field pea (*Pisum sativum*), lupin (Lupinus), faba/broad bean (*Vicia fabae*), lentil (*Lens culinaris*) and mung bean (*Vigna radiata*), have gained importance due to their major production [32]. India is the leading producer worldwide, followed by Canada, Myanmar and China. Notably, they are enriched with protein up to 30% of their weight on a dry basis), carbohydrates, dietary fiber, energy, important bioactive compounds and essential minerals and vitamins required for human health [34]. Among the pulses, lentils and beans have the highest Fe and Zn contents. The Fe and other minerals content are generally highest in beans. However, the presence of phytate and other inhibitors lower the digestibility or bioavailability of nutrients; thus, its removal is necessary. After its degradation, pulses would become potential sources of Zn and Fe due to immense mineral content [35]. The raw bean samples contained 0.49 mg vitamin B12 and 0.30 mg folic acid, although only 70–75% of water-soluble vitamins were retained in cooked seeds. The folate concentration of beans accounts for 95% of daily requirements. 

### 4.3. Oilseed Crops 

Oilseed crops are grown primarily for the extraction of oil contained in the seeds. Vegetable oils are used worldwide for household cooking and in other food products such as baked items and snacks. Apart from this, they are used as raw materials for various oleo-chemical industries. Oilseeds include canola, safflower, corn, sunflower, olive, soybean and peanut crops. As compared to other crops, oilseeds are considered a major vegetable oil source with a higher yield of oil [36]. In the semi-arid tropical regions, these crops are particularly important among low-income families as they contribute 40% of the total calorie intake in their diet. Recent reports confirmed the high protein and mineral-like Zn, Fe, Ca, etc. content in rape oilseed, thus making them the potential alternative to traditional crops for tackling nutritional stress [37]. Oilseeds are categorized into two classes known as temporary (groundnut, soya bean, castor bean, etc.) and permanent (coconut, oil palm, and olive) oilseed crops. Soybean, widely available as a meal, is enriched with amino acids and thus actively traded worldwide. Soy hulls are rich sources of minerals, fiber, protein and energy, with low-lignin content, and are highly palatable. Canola meal contains proteins, amino acids, vitamins and minerals with high sulfur content. Hemp seed and mustard seed oil are enriched with an adequate supply of nutritional parameters and several minerals including Ca, Mg, P, S, K, along with Fe and Zn. Likewise, other oilseeds are highly beneficial for human nutrition. Owing to the high oil content among oilseeds, major research is focused on brassica crops, which includes mustard, canola and rape, which enhances their importance in the bio-diesel industry for producing feedstock oils. However, the major constraint in oilseeds is the high level of phytic acid and other binding agents which reduce mineral bioavailability from the seeds. Therefore, major emphasis is given to developing the methodology for phytate removal to control or reduce the mineral binding in oilseed products. On the other hand, oilseed enriched with minerals via biofortification intervention may alleviate the mineral deficiency. The improved Se content in the plant components of oilseed rape has been observed on foliar application of Na_2_SeO_4_ under acidic location [38]. Limited research has been conducted on the biofortification of oilseed crops thus needs to be explored further to enhance their nutrition value.

### 4.4. Fodder Crops 

Humans depend on livestock directly or indirectly for their nutrition. Thus, nutrient deficiency in animals or their food will eventually affect the food of the end-user, i.e., human. Like humans, animals too need an adequate diet for optimal growth and productivity. However, plant food alone is insufficient in order to cover the gap of mineral intake; thus, the addition of mineral supplementation is required in animal feeds [39]. Globally, the forage grasslands used for animal feeding purposes have acquired 26% of the total land area and 70% of the agricultural area. Examples of forage crops include cowpea, sorghum, maize, Guar, Soybean, Berseem, Lucerne etc. In animals, two essential elements can be characterized as macro and microelements based on their daily intake required. Macro-elements include Ca, P and K, while Mn, Fe, Cu, Zn and Mo are part of the microelements category. Some non-essential plant elements are those such as Na, Se, Cr, I, Si and V, but essential elements for humans and animals make their way in plants via non-selective transport mechanisms, thus entering into humans and livestock food supply. The daily requirement of particular minerals for animals varies from humans, and also from one species to another. The presence of an adequate concentration of minerals and trace elements is important to control livestock health and also leads to minimal usage of antibiotics in animal-derived products. Recently, biofortification of fodder crops has gathered researcher attention due to their economic importance and agricultural trends globally [40].

A wide range of new cultivars involving *Medicago* spp., *Trifolium* spp., *Lolium*, and *Festuca* have been engineered to improve their biomass production, nutritional values, digestibility of DM, resistance against biotic stress (fungal and nematodes) and crop durability [41]. *Festulolium* has been developed by cross-breeding two closely related species of *Festuca* and *Lolium*, whereas backcrossing generated crops with more stable protein content in comparison to the parental line [42]. In another report, efficient biofortification has been achieved on temperate cultivars of *Lolium* and some tropical grasses including *Pennisetum* and *Brachiaria* [43]. Microbial biofortification has been achieved through the distinct sequence variants of fructan, as follows: Fructan 6G-fructosyltransferase increased the fructose levels in *Lolium* in hot climates. Promoters such as RBCS and chlorophyll a/b binding (CAB) generated the transgenic lines of *Lolium perenne* that eventually improved the yield, digestibility, fiber, and fructans content in leaf blades and pseudostems [44]. In *Medicago sativa*, a mutation has been successfully modeled in Squmaosa Promoter Binding Protein (SPL 9) gene using CRISPER/Cas9. Gao et al. revealed that advancement using the gene-editing approach for the improvement of fodder crops is limited [45]. Many genes have been identified which can be used to enhance forage crops. However, due to limited transformation technology, the development of new forage crops is lagging. Genome editing is a promising technology and will be more accepted in the future for forage crop development. Soil-applied Cu @ 6 kg ha^−1^ to oats fodder enhanced yield and Cu content due to increased bioavailability of Cu [46]. Sandhu et al. studied the biofortification of oats fodder with foliar-applied Cu. Foliar-applied Cu (0.2%) at 60 and 90 days of sowing significantly enhanced the Cu uptake, content, yield and protein content [47].

## 5. Biofortification through the Mode of Minerals Fertilization

**Figure 2:**
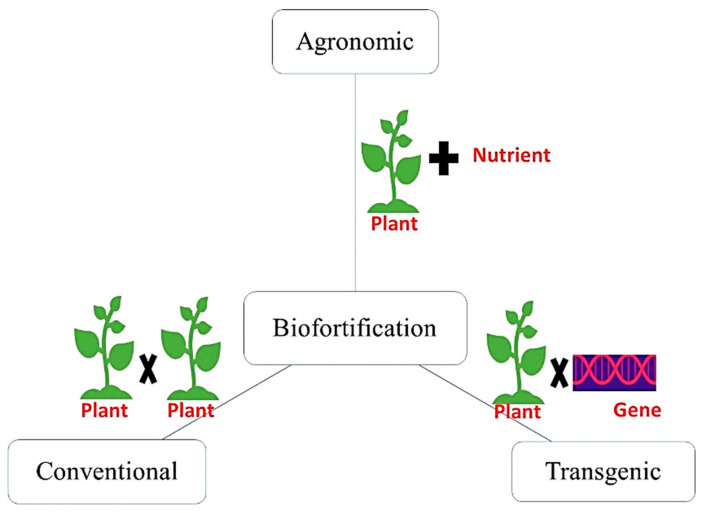
Different approaches to achieve biofortification.

Biofortification entails the improvement in the nutritional quality of a target crop by enhancing the micronutrient concentration in edible portions without sacrificing agronomic traits, i.e., pest resistance, yield, drought resistance [4]. Three major strategies to accomplish nutritional security through biofortification are conventional biofortification, transgenic biofortification and agronomic biofortification (Figure 2). 

The conventional approach refers to selecting existing varieties of high-yielding crops that naturally contain a higher content of nutrients of interest and cross-breed using conventional methods to produce staple crops with desirable nutrient and agronomic traits. Genetic biofortification can be attained through specific genetic manipulation to enrich micronutrient concentration in edible plant parts. Non-genetic measures or agronomic biofortification to enhance the micronutrient level in food plants could be more efficient. Indeed, this approach not only improves yields but also the nutritional quality of grains [6]. Kumar et al. reported the agronomic intervention as a sustainable and cost-effective approach to improve plant growth, yield contributing traits and Zn, Fe content in paddy crops using N, Zn and Fe fertilization [48].

**Figure 3:**
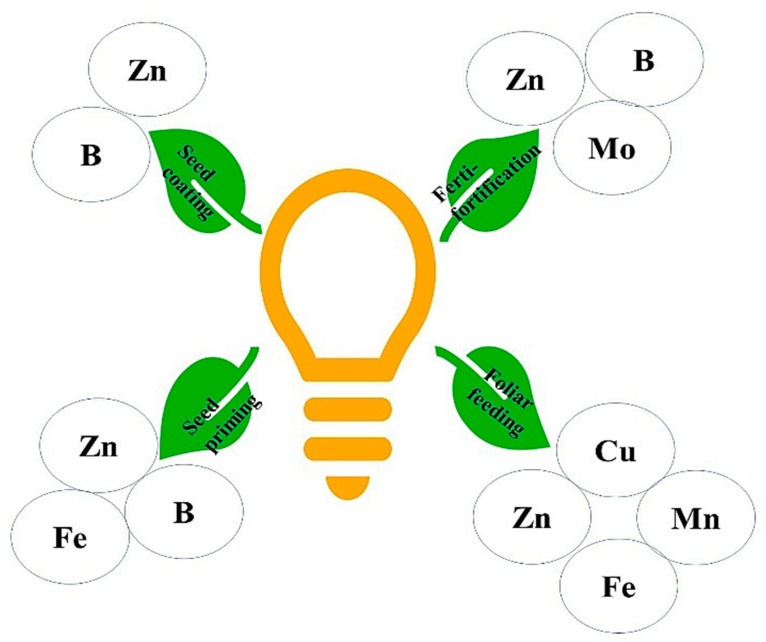
Different modes of mineral fertilization.

Agronomic biofortification refers to the micronutrient fertilizers applied to the soil and/or plant to enrich the edible part of the field crop with micronutrients, as shown in Figure 3 [6]. To date, numerous biofortified crops including cereals, pulses, oilseeds, fodder crops, etc. have been produced worldwide. During this process, the desired nutrient is added externally before or during the growth of plants. Soil application ensures a sufficient level of nutrient for root uptake, whereas foliar application increases the nutrient content in the leaf for its transportation to other plant parts. Soil properties such as pH, its calcareous nature, etc. affect the availability of the micronutrient and thus the success rate of biofortification. Thus, fertilization application mode and soil properties, along with amendments, affect the mineral enrichment in a particular crop.

### 5.1. Ferti-Fortification or Soil-Applied Fertilizers

Owing to multiple benefits, micronutrients application through soil is the most versatile and effective method, particularly to correct the deficiencies of B [49,50]. To alleviate Zn deficiency in different crops, soil application of ZnSO_4_·7H_2_O (21% Zn) at 62.5 kg ha^−1^ or ZnSO_4_·H_2_O (33% Zn) at 40 kg ha^−1^ have been found equally efficient and economical. The positive impact of Zn soil application on yield and Zn content in wheat and rice grain has been reported [50,51]. Soil Zn application has also been evidenced to affect the cowpea and chickpea in terms of yield and nutritional quality [52]. In different lentil cultivars, soil Zn application accounts for the grain production, plant growth, Zn concentration and bioavailability and reduced phytate concentration [53]. In soybean addition of Se and Zn to soil synergistically enhanced the Se and Zn content in seeds and subsequently improved physiological status [54]. The data on soil application of minerals has been represented in Table 1.

### 5.2. Foliar Feeding

Soil fertilization can enhance the micronutrient levels within the grain; however, it limits the salt intake and is not efficient for immobile minerals. Moreover, the micronutrients such as Fe present in the fertilizer become futile for soil application, as soluble Fe is readily converted to insoluble Fe^3+^ form, thus making it unavailable to plants. Thus, foliar feeding of fertilizers was implemented to overcome such problems. This intervention involves the fertilizers spray in the liquid state applied over the leaves. A single or mixture of micronutrient solutions in combination with the target salt is applied as a spray on the leaves where they are absorbed via stomata and epidermis. Thus, in particular cases, foliar feeding evidenced more efficient as compared to soil applications for effective use of nutrients and minimizing the visual deficiency and soil deficiency problems shortly. To date, a wide range of studies has been reported on foliar feeding of mineral fertilizers to different crops (Table 2). 

Under field conditions, foliar fertilization of Zn improves its concentration of inedible parts [6]. Four foliar sprays @ 0.5% of Zn and Fe to different growth stages of wheat cultivars enhanced grain Zn and Fe concentration to 17.3–38.8% and 13.1–30.3%, respectively, as well as grain yield to 2.5–5.1% [55]. Likewise, significantly improved grain Zn (30.8–44.8%) and Fe (22–38.2%) concentration, as well as grain yield (6–10.3%) through foliar application of Zn and Fe at different growth stages for rice and brown rice over control, has been reported [56]. In contrast, foliar-applied Mn and Cu enhanced grain Mn and Cu content, respectively; however, no significant increase was observed in the grain yield of wheat cultivars over control. The trend was attributed to the higher absorption of micronutrients through foliage [57]. Ferti-fortification of Fe (FeSO_4_·7H_2_O with 0.5% and 1% levels) in different rice cultivars at different growth stages increased the Fe grain concentration. The results supported the fact that owing to the genetic differences in different rice cultivars, there exists a difference in plant ability to acquire and sequester minerals. Further, the grain yield and Fe level in grain improved over control in paddy grains, brown rice and polished rice by foliar Fe application at different growth stages [58]. In diverse maize cultivars, foliar Zn and Fe fertilization at different stages increased the grain Zn concentration and yield traits depending on their absorption efficiency [59]. In different wheat cultivars, grain yield showed the trend aestivum > durum > control to foliar-applied Zn, Mn and Fe. The fertilizer application also enhanced Zn and Fe content in grain. Ram et al. reported superior results with foliar Zn application in wheat and rice over the soil Zn application [51]. The yield-related parameters and Zn concentration in rice were found to be higher in the case of integrated Zn application along with NPK, as compared to the sole Zn application. The results demonstrated that better availability of nutrients facilitates the nutrient uptake by roots and thus leads to better grain yield. Additionally, foliar-applied Zn and Mn enhance the crop yield including wheat, rice and barley [60]. In oats fodder crop, foliar-applied Cu at 0.2% at 60 and 90 days of sowing enhanced maximum Cu concentration and crude protein yield due to increased Cu uptake over control [47]. In another report, foliar-applied Fe improved the yield and Fe concentration along with other digestibility parameters in teosinte. Foliar fertilization also proved economical due to the improved net field benefit and benefit–cost ratio of teosinte [61].

In chickpea, soil (sowing) and foliar application of Zn followed by foliar application of urea (flowering and pod formation) improved plant growth and yield attributes, symbiotic parameters, productivity and profitability of crops. Urea enhanced the N content of plants during reproductive phases, thus facilitating protein synthesis and photosynthesis, and thereby improving yield and growth parameters [62]. In addition, in oats fodder, soil + foliar application of Zn enhanced the Zn accumulation in grains, biomass yield, crude protein content and fodder quality. Soil application of Zn has been proved beneficial to increase the availability of Zn in the rhizosphere, whereas foliar spray elevated the absorption and rapid translocation to storage organs. The increase in crude protein content, due to high Zn content attributed to the increased N-metabolism, thus enhanced the rate of protein synthesis due to the accumulation of amino acids. Zinc application through soil and foliar enhanced the Zn concentration in plants, which is associated with RNA and ribosome induction, the result of which accelerates protein synthesis [63]. In cowpea, soil application followed by foliar Zn application showed tremendous results in terms of yield and nutritional parameters over the sole soil or foliar application [64].

**Table 2:** Foliar application of micronutrients for mineral fertilization.

Micronutrient	Crop	Reference
Zn and Fe	Wheat	[55]
Zn and Fe	Rice and brown rice	[56]
Mn and Cu	Wheat	[57]
Fe	Rice	[58]
Fe and Zn	Maize	[59]
Zn	Wheat and Rice	[51]
Zn + Mn	Wheat, rice, barley	[60]
Cu	Oats	[47]
Fe	Teosinte	[61]
Zn	Oats	[63]
Zn	Cowpea	[62]

### 5.3. Seed Priming

Seed priming involves controlled hydration of seeds that permits them to perform their pre-germination metabolic events without radical emergence. Farooq et al. [65] reported that primed seeds have immense potential to give uniform stand establishment and productivity than dry seeds (Table 3). Seed priming of wheat and rice indicated better growth and yield [66]. Seed priming with Zn + *Pseudomonas* sp. MN12 was recorded as being more highly cost-effective than other modes of applications for bread wheat. [67]. Seed priming of wheat and barley with 2 mg L^−1^ each of Fe and Zn improves the grain yield and tillering of bread wheat demonstrating a beneficial and alternative approach for bread wheat fortification [68]. Seed priming with B content of 0.001% or 0.1% enhanced the stand establishment, whereas 0.5% content hinders germination [63].

### 5.4. Seed Coating

Seed coating involves the application of nutrients in the form of finely ground powder to the seed surface along with the inert sticky material (e.g., Arabic gum). This technique affects the seed or soil at the soil–seed interface and ultimately influences the availability of soil-applied and coated nutrients. The benefits of seed coating include uniform application of micronutrient source and reduction in the adverse effect on non-target pests, thus minimizing the environmental side effects. Moreover, the combination of treatments can be applied more precisely. Numerous factors including the nutrient:seed ratio, coated micronutrient, soil type, soil fertility, soil moisture and material used for coating alter the proficiency of micronutrients. Zn seed coating has been widely exploited in crop yield improvement parameters of various field crops. In comparison to non-coated seeds, Zn seed coating using ZnSO_4_ and ZnCl_2_ augmented the Zn concentration in tissue, germination and seedling growth in wheat crops [69]. Boron Seed coating (1.0, 1.5, 2.0, 2.5 and 3.0 g kg^−1^) of rice accounted for the improved uniform germination and tillering due to water relation and assimilate partitioning, ultimately B enrichment in rice grain [70]. Also, application of B through seed coating along with seed inoculation with micro-organisms proved to be viable option to enhance grain yield nodulation, growth and B level in chickpea. Coating with Mn resulted in better grain and straw yield, Mn uptake ratio and grain Mn concentration in wheat seeds [60]. 

## 6. Biofortification through Agronomic Management Practices 

Management practices are primarily focused on improving the soil condition that ultimately enhances the micronutrient availability for plant uptake and thus its concentration in food. Various factors such as physical, chemical and biological characteristics of soil determine the nutrient use efficacy of the plant. Well-known management practices are integrated soil fertility management (IFSM), tillage practices and water management. The integrated soil fertility management approach includes the use of mineral fertilizer, organic inputs and improved germplasm [71]. Apart from the soil organic matter sustainability, organic compounds advocate multiple benefits to soil status, such as water holding capacity, cation exchange capacity and soil structure. However, they release nutrients in the rate-determined manner, thus seldom meeting the crop nutrient requirement at the adequate time for optimum yield. Thus, sole usage of organic inputs and mineral fertilizers are not sufficient to cover the gap between mineral deficiency and the requirement of grain crops. Thus, the combination of both interventions is effective as they have positive interactions and complementary functions [72].

**Figure 4:**
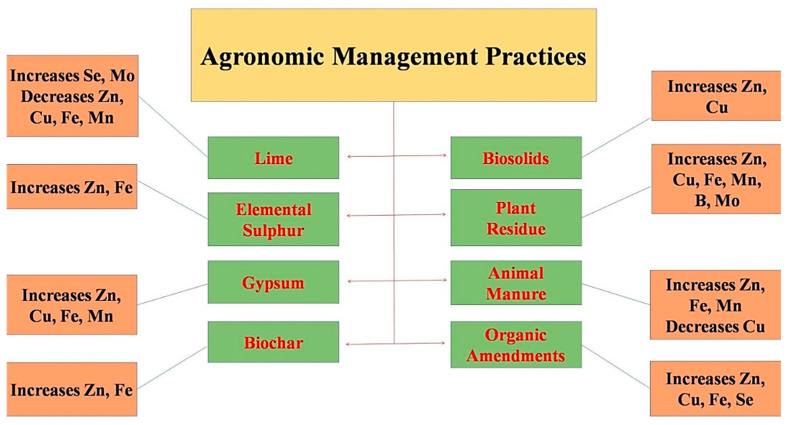
Different amendments under agronomic management practices to enhance mineral content in plant.

By nature, ideal soil for plant growth exists rarely worldwide; thus, major emphasis has been given to ameliorating the soil status, particularly chemical nature. With the increase in agricultural activities, various approaches for soil amendments have been frequently used and proved beneficial (Figure 4). Among the chemical soil properties, the soil pH plays a pivotal role in affecting plant growth, improving nutrient availability and eradicating harmful toxic substances. Thus, optimizing soil pH is a prerequisite to accomplishing quantitative and qualitative crop production. It involves either increasing the acid soil pH or lowering the pH of alkaline/calcareous soils.

### 6.1. Application of Lime

Acidic soil (pH < 7) refers to the presence of an excess of H^+^ ions and lack of basic cations. Thus, external supply of hydroxides, carbonates (lime) and other basic acting substances are effective approaches to neutralize soil acids. The soil-pH regulates the micronutrients solubility in soil; thus, a small variation in pH prominently affects solubility and uptake efficiency of micronutrients by plants. Literature studies have demonstrated the effectiveness of frequently applied limestone to ascertain crop productivity in acidic soils [73]. Further, limestone addition may reduce the uptake efficiency of Fe, Zn, Cu and Mn micronutrients; thus, this may lead to more severe micronutrient deficiencies [73]. Several studies have inspected the effects of P fertilization in association with lime. P shows negative interactions with Zn and adversely affects its availability in almost all soils, thus aggravating Zn deficiency in plants, with corresponding decreases in grain Zn concentration. Thus, Zn fertilizers must be applied along with limestone application to eradicate Zn deficiency. Soltani et al. also observed the positive interactions between lime and Zn, which increased grains and straw yield more than two-fold as compared to the control [74]. Zn application boosted several metallic enzyme activities, auxin production and regulatory functions, thus enhancing carbohydrates synthesis and their upward movement to grain production and filling sites in the rice plant. On the other hand, the addition of lime alters soil properties to make it more suitable for rice growth.

### 6.2. Application of Elemental Sulfur

The significant enhancement in grain Zn content through S and Zn has been reported in the literature, whereas sole Zn application to soil may generate nutritional disorder due to the antagonistic effect of Fe and Zn. Thus, combined application of both minerals was recommended for the nutrition of wheat crop, while considering soil properties such as pH, texture, electrical conductivity, organic matter, CaCO_3_, S and N of soils and cultivated plants. Significant improvement in mineral concentration and nutritional value of rice grain has been observed by S application along with Fe and organic input (biochar manure/poultry manure) as compared to lack of S application. Oxidation of S reduced soil pH and thus enhanced the solubility and uptake of Fe in rice grain, whereas Fe application reduced phytate content. Biochar facilitates the microhabitat for microbes to Fe-chelating compounds that regulate its mobility towards the grain. Thus, the synergistic effect of all the components enhanced the grain Fe content [75]. 

### 6.3. Application of Gypsum (CaSO_4_·2H_2_O)

Alkaline soil (pH > 7) is generally associated with high amounts of carbonates and are calcareous. Management practices such as deep ploughing increase the soil pH by increasing the amount of carbonate in the surface layers, and are thus avoided. Further, excess amounts of Ca and Mg in calcareous soils eventually lead to multi-nutrient deficiencies, as elevated soil pH reduces the solubility of Fe and Mn. Acid-producing amendments such as gypsum and elemental S have been widely used for amending sodic and saline-sodic soils to decrease soil pH [76]. Gypsum facilitates the removal of excess exchangeable Na with Ca from the soil cation-exchange complex and bicarbonate from the soil solution. Removal of bicarbonate lowers the soil pH and consequently increases plant-available Zn, Fe, Cu, Mn and Co. The trend attributed to the decrease in soil pH and improved soil physical properties via gypsum application. Higher uptake of nutrients in maize has been recorded with the application of slag-based gypsum @ 750 kg ha^−1^ in acidic and neutral soils [77].

### 6.4. Application of Biochar

Application of biochar to acidic soils may enhance the micronutrient uptake as well as plant growth and thus may prove as an effective measure for waste materials disposal, while improving soil fertility and crop quality. The potential benefits of integrated application of biochar and *Burkholderia phytofirmans* PsJN have been observed for Fe bioavailability, yield and nutritional value of quinoa grains under iron-limited saline conditions. Thus, siderophore-producing microbes with organic amendments improve Fe grain concentration, and reduce antioxidants activity along with the Na+/K+ ratio of *C. quinoa*. Further, combined application of Zn + biochar showed superior results to improve crop yield and grain Zn concentration, while reducing grain Cd concentration, over sole Zn and sole biochar application [78]. Integrated use of biochar and zerovalent iron (BZVI) showed a positive impact on Zn and Fe concentration in rice, and reduced the concentration of Cd by 83% as compared to the untreated field. Biochar may facilitate Cd sorption and enhanced ZVI corrosion by-products, while sole application of ZVI and BC reduced Cd and Fe concentration. BZVI improved the Fe concentration due to Fe^2+^/Fe^3+^ released from BC-enhanced ZVI corrosion, whereas Zn concentration was improved due to competitive sorption of Cd^2+^ on BZVI, which facilitated the desorption and translocation of Zn [79]. Foliar ZnO NPs application alone or in combination with biochar improved the plant growth parameters, chlorophyll concentrations and Zn concentration in maize. The combined application reduced the Cd content, electrolyte leakage, MDA and H_2_O_2_, whereas it improved the antioxidant enzyme activities over the control. The reduced Cd concentrations might be attributed to the competitive Zn uptake due to ZnO application through roots and increased biomass. Biochar alters the soil pH and thus changes the Cd fractionation in the soil to reduce the bioavailability of Cd and other metals in the soil. 

### 6.5. Application of Biosolids

Biosolids contain essential nutrients including N and P as well as soil organic matter. Sewage sludge is also a source of numerous metals, particularly Zn, at varying concentrations, depending on the source. Application of sewage sludge for at least 2–8 years increased grain Zn content of wheat. The increased Zn content was associated with the increased soil Zn content provided by sewage sludge. In a pot experiment, the impact of sewage sludge at doses of 10%, 20% and 30% of fresh soil weight on the nutrient content of sunflower and maize was evaluated. Fertilization with sewage sludge significantly increased the Zn content in the soil and plants over control [80]. The combined application of lime and municipal sewage sludge (15%) resulted in a two-fold increase in the Cu concentration and a three-fold increase in the N_2_ concentration. Copper speciation analysis showed that the municipal sewage sludge contains the highest amount of Cu in the organic and residual fractions with the little amount present in easily soluble and exchangeable fractions bound to amorphous iron oxides. Application of sewage sludge released Cu into the soil and decreased the amount of the residual fraction [81]. The sewage sludge addition also increased the Zn accumulation in rice.

### 6.6. Incorporation of Crop Residue

Developing countries produce more than 1000 million tons of cereal residues annually. Wheat, rice, corn, barley, soybean, potato and rapeseed are the major residue-producing crops globally. Crop residues are a good source of several micronutrients among which only rice and wheat remove 777, 745, 96, 42, 55, and 4 g ha^−1^ of Fe, Mn, Zn, Cu, B, and Mo per ton, respectively. Recycling of crop residue assists the significant recovery of micronutrients, thus improving their soil availability. About 50% to 80% of Cu, Zn and Mn used by rice and wheat crops can be recycled through residue addition. Under rice–wheat rotation, the addition of cereal straw along with ZnSO_4_ increased grain yield, Zn concentration in plant tissues and total Zn uptake by crops, due to enhanced bioavailability of Zn [82]. Incorporation of preceding clover crop residue to wheat crop enhanced protein density, Zn concentration in grain and straw without compromising the yield traits. The results attributed to enhanced N supply from clover treatment and formation of soluble Zn complexes with increased concentrations of organic ligands in the soil solution from precrop plant [83]. Additionally, abiotic factors also influence plant growth and thus plant nutrient uptake. Thus, crop residue management strategies must include understanding of crop residues interactions with nutrients recycling as well as with soil physical, chemical and biological properties, and crop production. 

### 6.7. Application of Animal Manure

Traditional nutrient management practices include the use of livestock manure as it is a good source of plant nutrients and can also alter the physical and biological properties of the soil. Manure was found to be a better source of available Zn, Fe and Mn as compared to synthetic fertilizers, but accelerated the depletion of available Cu as it may bind to organic matter. On the contrary, no significant effect was observed on available Cu due to applied manure, possibly due to the low amount of Cu manure used. In addition to Zn supply to the soil, manure also promotes chemical and biological reactions that result in the dissolution of non-available Zn. Cattle manure along with forest leaf litter and Zn-enriched fertilizer resulted in massive increases in maize grain yield and grain Zn concentration. Long-term application of organic matter to the soil not only increases the total Zn content of the soil but also the proportion of labile Zn, which is the readily available form for plant uptake [52]. The combined application of FYM and lime to maize grown on alfisols evidenced the increase in the concentration of extractable Zn and Zn uptake in acid soil due to a lowering in soil pH [84]. Further, application of FYM along with crop residue and lime has significantly improved the nutrient status of soil, whereas foliar-applied Zn + Fe enhanced the Zn and Fe content of rice grain up to 53% and 38.34%, respectively, in acidic soil. Zinc fertilization, along with cattle manure and woodland leaf litter, enhanced the grain Zn concentration in cowpea production under legume–maize rotational sequence, particularly in sandy soils [52]. The addition of livestock manure with a raised soil organic carbon concentration and cation exchange capacity to organic farms reflected higher soil and wheat grain Cd concentration than conventional farms due to increased Cd binding sites. The increased soil Zn was due to Zn supplementation in animal feed subsequently in manure to soil [85]. Sulfur and Zn fertilization along with farmyard manure enhanced the yield and nutrient uptake over sole nutrient fertilization in rabi mustard [86]. Subsequent use of S and Fe along with poultry manure lower the pH, phytate and polyphenol content while increasing the grain Fe and ferritin concentration in wheat grains in calcareous soil. The results attributed to the synergistic effects as S lowers the soil pH thus improving Fe solubilization and thus its bioavailability in alkaline soil.

### 6.8. Addition of Compost

Composts production is greener and more economical in urban and rural areas, thus are found to be inexpensive and effective Zn and Cu fertilizers. Municipal compost and green waste compost enhanced grain yield and grain Zn concentration (220% higher) of open-pollinated variety over commercial maize variety with the lower accumulation of heavy metals (74% lower nickel concentrations) [87]. Long-term application of compost increased soil organic carbon, pH and cation exchange capacity while reducing DGT-available Zn and Cd concentrations over control. Different Zn and Cd uptake behavior by wheat increased the Zn/Cd ratios of wheat grains and thus quality parameters by reducing Cd accumulation. Zn-enriched compost with Zn solubilizing bacteria (Zn-EC60:40) showed better growth and yield parameters of rice through a slow and steady release of Zn from ZnO over ZnSO_4_. The treatment also enhanced Zn accumulation in various parts of rice under Zn deficient conditions thus significant under field conditions at various places. The major constraint for the application of compost is the potential presence of heavy metals, pathogens, xenobiotic compounds, and element imbalance. Thus, before the application of compost, its interaction with other parameters is studied. 

## 7. Selection of Suitable Crops and Cropping Systems

Generally, the system of cropping affects soil characteristics and thus influences micronutrient availability. Precrop residual effects of root litter on soil physical and chemical parameters, as well as micro-organisms and other plant-soil interactions, affect the quality and quantity parameters of succeeding crops. Augmented soil physical properties assist better the root growth conditions and thus increase root uptake of immobile micronutrients.

### 7.1. Suitable Crops 

Prior to the cropping system, the selection of suitable crops to alleviate nutrient deficiency and other constraints is mandatory. Crop rotation patterns should be planned using crops that complement each other. For example, cereals are complemented by legumes, e.g., in the case of the brassicas–legumes system, the latter assist in nitrogen fixation, whereas the former is beneficial as a nutrient-rich source. The example of the intercropping system includes three sisters, i.e., corn with climbing beans and squash or pumpkins. Corn provides a stalk for beans to climb, while beans improve the nitrogen status for N-fixation. The squash provides a shield for weed suppression and preserves soil moisture. Also, a nitrogen-fixing crop should always precede a nitrogen-depleting one, and a low residue crop should be followed by a crop having high biomass. As observed by Diekow et al., higher residue input from legume-based cropping systems significantly enhanced soil organic carbon. Further, long-term planting of legumes as a winter cover crop resulted in higher soil organic carbon levels compared to the mono-cropping system [88].

### 7.2. Suitable Cropping System

Literature studies revealed the significant impact of cropping systems on the qualitative and quantitative traits of the plant. As observed, cropping systems affects the concentration of phyto-available Cu in soil and decrease in the following order: continuous clover > crop–legume rotation > continuous wheat due to variable Cu uptake capacity of each crop. Evidently, incorporation of clover residue as a preceding addition into the soil enhanced the accumulation of grain Zn and N, with a simultaneous decrease in the phytic-acid-to-Zn (PA:Zn) molar ratio in a subsequent wheat crop on a Zn-deficient calcareous soil. Clover improved soil N content via fixation of atmospheric N and enhanced Zn availability and accumulation, owing to the formation of soluble Zn complexes with organic ligands released into the soil from the pre-crop plant [83]. *Pseudomonas* secrets Fe chelating siderophores thus improves Fe nutrition and growth under Fe-limited conditions [89]. Likewise, soybean-maize intercropping with alternating organic fertilizer and strips enhanced Fe concentration in soybean [90]. 

## 8. Biofortification through Breeding Approaches

Biofortification through soil or foliar interventions can improve the nutrient density in phloem-fed tissues, such as seeds, fruits and tubers [6]; yet, restricted mobility of nutrients in phloem limits their impact. As in the case of Zn, its limited mobility confines its accumulation in the edible plant parts. Conventional breeding approach along with advanced biotechnological methods and phenotyping as emerged as a great tool to enhance the micronutrient level in new cultivars of staple crops. This approach practices crossing together plants with the desired combination of characteristics without sacrificing agricultural and economic traits. The study of the genetic basis of the micronutrient accumulation in food grains along with the mapping of the quantitative trait loci form the basis of plant-breeding strategies to improve yield and micronutrient content in grains through marker-assisted selection.

### 8.1. Screening of Crop Cultivars

Nutrient efficient genotype can be described as a genotype that produces more economic yield under identical conditions with a given quantity of applied or absorbed nutrients than other genotypes. The nutrient efficiency ratio can be defined as the amount of biomass produced per unit nutrient absorbed and is equivalent to the reciprocal of plant nutrient concentration. Micronutrient efficiency of specific plant cultivar can be calculated using the following equation:

(1) $$\mathrm{Micronutrient}\;\mathrm{efficiency}=\frac{\mathrm{Yield}-M}{\mathrm{Yield}+M}\times 100$$

where −M is micronutrient deficiency and +M is micronutrient fertilization.

The sole use of fertilizers to alleviate micronutrient deficiency is not worth it from an agronomic, economic, and environmental point of view. The use of micronutrient-efficient crop cultivars results in more yield and micronutrient concentration in low phyto-available micronutrient contents, thus diminishing the use of fertilizer inputs and improving crop nutritional quality on low fertility soils. Numerous crop cultivars have been investigated for their germplasm variation for nutrient efficacy from the soil [91]. Various factors responsible for nutrient efficiency are bioavailability of nutrients through root processes, root uptake and translocation of nutrients, altered subcellular nutrient compartmentation in shoot cells and biochemical utilization of nutrients. Genetic variations among cultivars have been observed as a major factor that controls the nutrient response of particular plant species. The detailed knowledge of genetic variation facilitates the development of more efficient plant genotypes through increased root uptake, translocation and utilization efficiency. Indeed, the adaption of this intervention is beneficial to achieve food and nutritional security in developing countries. For instance, Fe and Mn deficiency stress in plants growing on alkaline or calcareous soils due to their rapid conversion to immobile forms is a widespread problem. Thus, screening of Fe efficient genotypes of numerous crops has been beneficial for correcting Fe deficiency. Likewise, zinc-efficient genotypes have been exploited for their immense tolerance under Zn deficient conditions than Zn-inefficient genotypes. The international genomes of wheat (*Triticum aestivum* L. and *T. durum* Desf.), rice (*Oryza sativa* L.), maize (*Zea mays* L.), cassava (*Manihot esculenta* Crantz), beans (*Phaseolus vulgaris* L.) and sweet potato (*Ipomoea batatas* L.) have been studied for micronutrient enriched traits [92]. 

Based on the grain yield efficiency index and nutrient uptake efficiency, genotypes can be categorized into four classes. The first category consists of efficient and responsive genotypes (ER) that can produce a higher yield and nutrient uptake efficiency than the mean value of the grain yield efficiency index and nutrient uptake efficiency index. The second category consists of those genotypes which were efficient and non-responsive (ENR), i.e., they can produce a high yield but low response to nutrient application, thus low uptake efficiency. Another group includes the inefficient and responsive (IER) genotypes having a low yield but high nutrient uptake efficiency index. Group 4 includes genotypes that are inefficient and non-responsive (IENR) having a lower yield and nutrient uptake efficiency index. The ER genotypes are recommended for farmers, whereas ENR and IER genotypes can be used by plant breeders for developing efficient and responsive genotypes by identifying the genes responsible for the responsive traits. Jhanji et al. reported that among 24 diverse wheat genotypes, 12 (PBW 636, HD 2967, PBW 621, DBW 17, TL 2942, PBW 550, BW 9184, BW 9179, BW 9149, SAMNYT 410, BW 8989 and SAMNYT 413) were found to be ER under low Mn supply [93]. For short-duration rice genotypes, among 15 genotypes 8 (CO 51, CO 47, ADT 37, ADT 36, MDU 5, TKM 12, MDU 6 and IR 50) were found to ER under low Zn supply [94].

(2) $$\mathrm{Zinc}\;\mathrm{uptake}\;\mathrm{in}\;\mathrm{grain}\;\left({g\;\mathrm{ha}}^{-1}\right)=\;\mathrm{Grain}\;\mathrm{yield}\;\times \;\mathrm{Grain}\;\mathrm{Zn}\;\mathrm{concentration}$$

(3) $$\mathrm{Yield}\;\mathrm{Efficiency}\;\mathrm{index}=\frac{\mathrm{Grain}\;\mathrm{yield}\;\mathrm{under}\;\mathrm{low}\;\mathrm{Zn}}{\mathrm{Grain}\;\mathrm{yield}\;\mathrm{under}\;\mathrm{high}\;\mathrm{Zn}}\times 100$$

(4) $$\mathrm{Uptake}\;\mathrm{efficiency}\;\mathrm{index}\;=\frac{\mathrm{Zn}\;\mathrm{uptake}\;\mathrm{in}\;\mathrm{grain}\;\mathrm{under}\;\mathrm{low}\;\mathrm{Zn}}{\mathrm{Zn}\;\mathrm{uptake}\;\mathrm{in}\;\mathrm{grain}\;\mathrm{under}\;\mathrm{high}\;\mathrm{Zn}}\times 100$$

Thus, screening of efficient and responsive genotypes in plant cultivars is an economical strategy to achieve food and nutritional security under stress conditions.

### 8.2. Conventional Breeding

**Figure 5:**
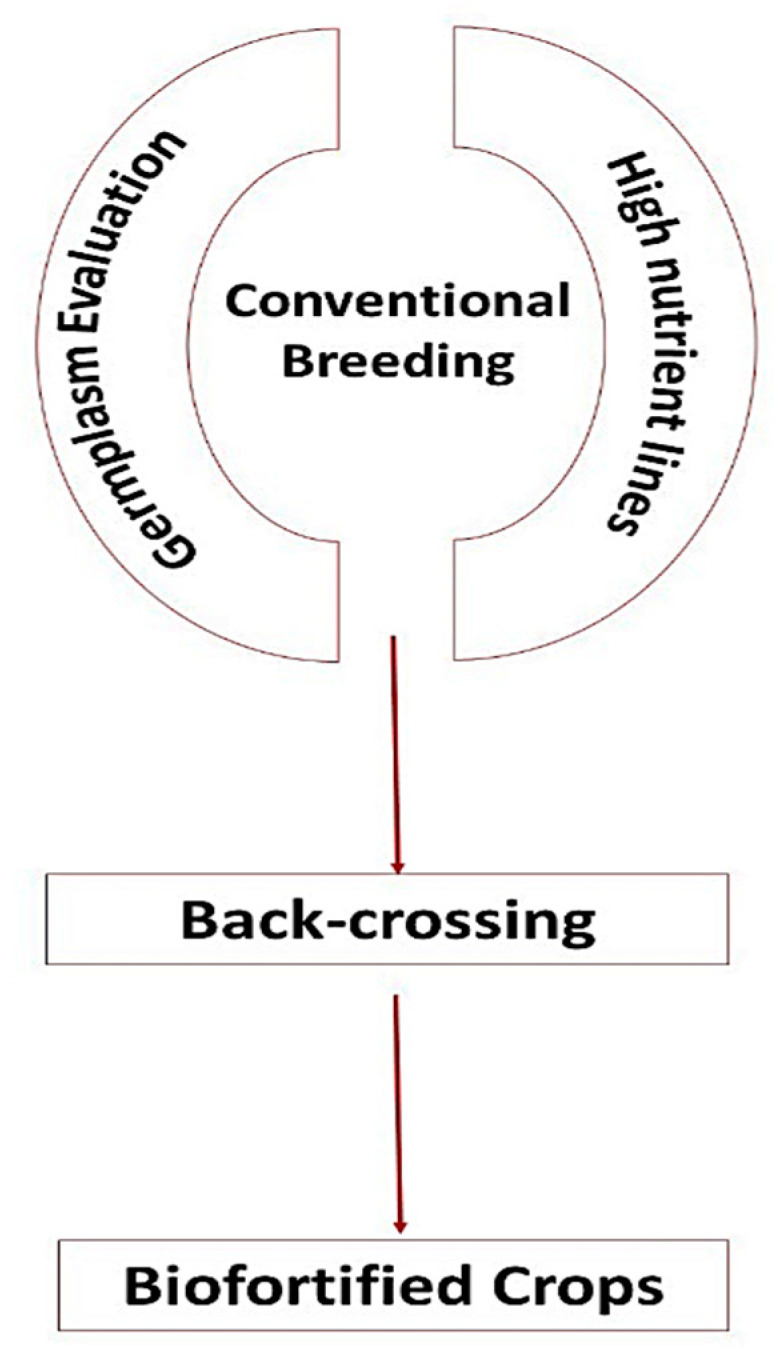
Conventional breeding approach to achieve biofortification.

Conventional breeding includes the crossing of available plant varieties having economical values with other varieties having high nutrient density lines (Figure 5). To date, two hundred and ninety varieties of biofortified crops have been developed using this technique and released for production in more than 30 countries, and hundreds more are being tested in over 60 countries. The new cultivars of the crops including iron bean and pearl millet, zinc maize, rice and wheat, vitamin orange sweet potato, yellow cassava and orange maize have been produced. These biofortified crops are developed to fulfil the dietary intake gap of zinc, iron and vitamin A among the global population, based on their usual eating patterns where these crops are consumed as staples. The released varieties have been approved officially by national committees, thus indicating the importance of breeding intervention to achieve nutritional security by increasing the micronutrient density in these crops. Orange-fleshed sweet potato has been conventionally biofortified with β-carotene contents of 30–100 mg L^−1^ over the local varieties having less than 2 mg L^−1^ [95]. Conventionally biofortified white maize possessed higher yield contributing parameters with better agronomic traits such as drought tolerance and disease resistance [96]. Genome-wide association studies have been employed to exploit the different genomic regions in maize for the biofortification of Zn and Fe [97]. 

Sorghum lines with genetic variability were exploited for grain Fe and Zn concentrations. Higher variance magnitude of specific combining ability than general combining ability for grain Zn and Fe concentrations reflected the role of the non-additive gene in the nutritional trait improvement. Hybrids displayed simultaneous heterosis for agronomic traits and grain Fe concentration with limited grain Zn concentration, implying that intra-population improvement for the micronutrients is likely to be highly effective. Thus, accessibility of these parental lines may prove immensely beneficial for breeding programs.

### 8.3. Transgenic Technology/Biotechnological Approach

The conventional breeding approach is widely accepted to increase the micronutrient concentration in crops. However, exploitation of transgenic approach for biofortification is necessary due to several weaknesses which involve the following: the absence of elite genotype for the desired trait within the species (e.g., provitamin A in rice), or incongruous varieties for conventional breeding (due to a lack of sexuality; e.g., banana), long time period required to introduce single or multiple traits (pyramiding traits) into locally adapted elite varieties, inability to target nutritional traits to specific organs and inadequate knowledge of QTL × environmental interactions. The existence of an inverse relationship between grain mineral concentration and grain yield also limits the application of conventional breeding. This arises the need for new gene-editing techniques such as transcription activator-like effector nucleases and increased availability of fully sequenced genomes in staple crops. The transgenic approach involves the synthesis of transgenes that causes the micronutrient re-translocation between tissues and enhance their bioavailability, increasing the efficacy and reconstruction of biochemical pathways. Various crop varieties have been biofortified with micronutrients using a transgenic approach. To improve Fe and Zn content in the crops, the major emphasis is to increase the uptake and utilization efficiency of plants through variation in transporters expression and suppressing the anti-nutrient (such as phytic acid) concentration. Genetically modified rice containing soybean ferritin genes and nicotianamine synthase resulted in a six-fold higher endosperm Fe concentration retaining grain yield and quality parameters [98]. In the case of rice, overexpression of sole IRT1 enhanced the iron content 1.7-fold and 1.1-fold in leaves and grains, respectively, whereas a four-fold increment was achieved in polished rice through the combination of IRT1 and PvFER1 in the endosperm [99]. The results suggested the Fe accumulation in the vegetative tissues was owing to the lack of extra sink capacity in the seeds on the sole application of IRT1. Furthermore, the combined overexpression of NAAT and NAS1 in rice resulted in a four-fold increase in Fe concentration [100]. In the case of vitamins, the adequate regulation of limiting step in the biochemical pathway of seed for the facile production of vitamin-A precursor, i.e., β-carotene or alternative pathway for amplified production is the widely accepted transgenic approaches. For Se transgenic approaches focused on (i) the conversion of selenate to selenite and SeCys to SeMet by APS and Cystathionine synthase enzyme, respectively, (ii) the avoidance of SeCys into proteins by enzyme SeCys methyltransferase (SMT) and (iii) Se volatilization.

## 9. Micronutrients Sources for Mineral Enrichment

The efficacy of foliar application to a particular crop is governed by the source of micronutrients used for biofortification. The nature of the compound used regulates the ease of nutrient absorption and burning effects on the leaf surface. For efficient absorption, highly soluble compounds should be used over sparingly or insoluble compounds. The addition of surfactants further increases the nutrient penetration through the leaf cuticle [101]. Furthermore, chelated fertilizers, although expensive, have shown more potential than non-chelated fertilizers. For Zn biofortification, Zn salt such as zinc sulfate (ZnSO_4_), chelated Zn sources such as Zn-DTPA, Zn-EDTA, Zn-arginine and Zn-glycine), Zn nanoparticles, and Zn nano-chelates have been used in foliar spray solutions. For Fe biofortification of cereal grains, numerous chelated and non-chelated Fe foliar sources have been used till date (Table 4).

### 9.1. Mineral Solutions

Foliar-applied ZnSO_4_ showed the highest efficacy in increasing the grain Zn concentration in rice, wheat and maize over control [6]. Other sources have shown similar or fewer effects for Zn biofortification in cereal crops. Montoya et al. showed the superior Zn biofortification in maize employing foliar ZnSO_4_ than Zn-chelates (mixture of chelating compounds DTPA-HEDTA-EDTA) with elevated N_2_O emission. The results were further improved with the use of N fertilizer (urea) along with Zn fertilizer [102]. For Se biofortification through the foliar application, two major sources namely sodium selenite (Na_2_SeO_3_) and sodium selenate (Na_2_SeO_4_) have been used. As observed, Na_2_SeO_3_ showed better results for rice grain Se concentration than Na_2_SeO_4_. On the contrary, the higher results for Se concentration in rice grain were achieved with Na_2_SeO_4_ application as compared to Na_2_SeO_3_ application [103]. Soil application of Na_2_SeO_4_ for Se concentration has evidenced the reduced soil availability and plant concentration of added SeVI with ageing time. Kinetic studies employing a reversible first order equation model reflected the most pronounced ageing in calcareous soils and increased with CaCO_3_ content. It may prove beneficial to apply Se fertilizers in small doses more frequently to optimize the efficiency of use and biofortification program [104]. Further, foliar-applied Na_2_SeO_4_ increased the cowpea yield and grain Se concentration over control. However, application above 50 g ha^−1^ increased oxidative stress due to increased lipid peroxidation rates and hydrogen peroxide concentration. Additionally, an immense increase in calcium ions was also observed in damaged areas thus reported as a sign of abiotic stress in plants. Foliar fertilization of iodine has been reported employing potassium iodide (KI) and potassium iodate (KIO_3_). Although both are found to be efficient for improving the grain I concentration in cereals, KI is more suitable for spraying concentrated I solutions [6]. Iodine biofortification employing KI enhanced I concentration in maize (90%), wheat (58%) and rice (57%) grains over control. Keeping in view of I volatilization as methyl iodide, and to increase its penetration from the leaf surface surfactants such as potassium nitrate, KNO_3_ are recommended [105].

**Table 4:** Micronutrients sources for mineral enrichment.

Source	Crop	Reference
ZnSO_4_	Rice, Wheat, Maize	[6]
Zn-DTPA, Zn-HEDTA, Zn-EDTA	Maize	[102]
N + Zn + Fe	Wheat	[106]
Zn, Mn, Fe, Cu and B	Wheat	[107]
Zn, I, Se and Fe	Wheat	[108]
Na_2_SeO_4_ + ZnSO_4_	Wheat	[109]
FeSO_4_·7H_2_O + ZnSO_4_·7H_2_O	Wheat	[110]
Cu + Zn + Mn + NPK	Winter-wheat	
ZnSO_4_ + Zn-HEDP	Wheat	[50]
EDDHA-Fe(III), EDTANa_2_Fe(II)	Rice	[111]

### 9.2. Multi-Micronutrients Mixtures

Multiple micronutrient deficiencies in field crops limit the application of a single nutrient to improve nutritional quality. Separate application of each micronutrient is not economically viable; thus, multi-micronutrient mixtures have been employed to achieve the target. Split application of N at 160 kg ha^−1^ at sowing and stem elongation, along with soil + foliar application of Zn and Fe were employed to increase grain yield and protein, as well as grain Fe and Zn concentration [106]. Foliar feeding of multiple micronutrients (Zn, Mn, Fe, Cu and B) improved the nutrient density, growth and yield contributing parameters of wheat flour over control [107]. Zou et al. demonstrated that foliar mixture comprising of Zn, I, Se and Fe enhanced the Zn, I, Se and partly Fe concentration without yield trade-off in wheat under variable management and environmental conditions in six countries [108]. A foliar spray, containing a mixture of Se (Na_2_SeO_4_) and Zn (ZnSO_4_) enhanced plant growth as well as grain Se and Zn content in wheat with reduced Cd uptake under Cd stress [109]. Further, foliar application of Zn (0.3%) in conjunction with Fe (1%) improved yield traits in wheat with the highest increase in flagging to grain filling stages application. However, maximum Zn and Fe content was achieved with Zn and Fe application at rate 0.4% and 2%, respectively [110]. The multi-micronutrient mixture containing Cu, Zn and Mn along with NPK fertilization, improved Cu and Zn content in winter wheat grain (22.6% and 17.7%, respectively). 

### 9.3. Chelates and Chelating Agents 

Chelates, organic compounds, consist of polydentate ligands bonded to a central metal ion. The benefits of chelates over other sources include the following: the requirement of lower quantities, as they are completely assimilable by crops; facile absorption by plant roots or leaves; the removal of positive charge from the micronutrients facilitates the penetration through the negatively charged pores on the leaf and root surface more rapidly, else there is fixation of positively charged micronutrients at the pore entrance. Chelates are easily translocated within the plant and are not readily leached from the soil due to their adsorption on the surface of soil particles. Dhaliwal et al. observed that soil application of Zn (ZnSO_4_) followed by foliar-applied Zn-HEDP (C) increased the grain yield, grain Zn concentration and other plant growth parameters in PBW550 wheat variety, particularly in Zn-deficient sandy loamy soil [50]. Literature studies indicated that the use of FeSO_4_ in foliar spray has significantly elevated the grain Fe concentration in cereal crops. The addition of surfactant in the spray solution along with Fe-chelated source relatively enhanced grain Fe concentration. Chen et al. observed that as compared to EDDHAFe(III), EDTANa_2_Fe(II) increased Fe grain concentration along with better mitigation of Cd accumulation in rice via formation of the EDTANa_2_Cd complex in solution. The complex formed decreased the net Cd influx and increased net Cd efflux in root micro-zones and inhibited genes expressions involved in the transportation of Cd and Fe [111]. As chelates release micronutrients in a time-dependent manner, the kinetics of micronutrient release in soil application can be studied through various kinetic models.

## 10. Biofortification through Nano-Technology

Nanotechnology, a multidisciplinary field, enables characterization and selective manipulations of matter at the nanoscale to exhibit superior characteristics to its bulk counterpart. Thus, applications of nanomaterials have led to astonishing discoveries in important human endeavors, including agriculture, pharmaceutical, cosmetics, materials science, electronics and communication, waste-water treatment and environmental remediation, enzyme mimics etc. Moreover, the properties of nanomaterials can be altered by tailoring their composition [112]. The high surface area, increased penetrability, controlled release and target delivery of the desired nutrient to the plant system through ENMs has provided a promising platform in the area of agriculture over conventional modes. Apart from the sole application of ENMs to plants, ENMs incorporated into conventional fertilizers/pesticides have been exploited for their role in plant growth, disease suppression and nutritional as well as yield enhancement. Till now, numerous ENMs including metallic oxides, metalloids or nonmetals have been employed in agricultural practices with varied outcomes based on plant species, dose, application mode, experimental/exposure design and environmental conditions (Table 5). 

Importantly, the fate and effect of employed ENMs on the environment and human health must be evaluated prior to practical implementation at a large scale. The studies indicated that phytotoxicity of ENMs is concentration-dependent as some ENMs are phytotoxic at higher concentrations, but at lower concentrations beneficial effects were more evident.

### 10.1. Micronutrients Nano Fertilizers 

Recent studies showed the importance of nano foliar application on plants nutrition and their protection. Apart from foliar plant protection employing silver NPs and TiO_2_, numerous nutrients have been applied through foliar spraying, such as nano-selenium, nano-silica, CuO NPs, sulfur NPs, nano Zn, Fe_2_O_3_ NPs etc. The exceptional properties of NPs, including their large surface to volume ratio, high surface reactive capabilities as well as electron exchangeability, confirmed their importance under different challenging environments. Zinc oxide NPs possessed potential benefit over ZnSO_4_ due to less toxic responses to wheat crop at high concentrations, while increasing the grain yield and grain Zn concentration [113]. Iron oxide NPs have been used for Fe biofortification with alleviated Cd content in wheat by using Fe NPs. The results attributed to the fact that the regulation of Cd ions into the plants is monitored via metal transporters such as Fe transporters. Thus, the external supply of Fe may interfere with Cd uptake via roots [114]. Keeping in view of plant leaf structure (pore sizes in cell walls) the size of the NPs to be applied is devised. The transfer of NPs greater than the size of leaf pores will be hindered. Different NPs applied to the leaf surfaces enter either through the stomata pores or through trichrome bases for their translocation in various plant tissues. Graphene oxide has been exploited for its potential as a nanocarrier loaded with micronutrients (Zn and Cu) in a pot trial. Kinetic studies demonstrated that the prepared fertilizers showed a biphasic dissolution behavior relative to commercial fertilizer granules with fast- and slow-release micronutrient release. The results demonstrated that Zn and Cu uptake by wheat was higher when using GO-based fertilizers over standard zinc or copper salts. Thus, this study explored the potential of GO as a nutrient carrier for field experiments [115]. Recently Zinc hydroxide nitrate (Zn_5_ (OH)_8_(NO_3_)_2_·2H_2_O) has been exploited for its potential as a foliar spray solution. The novel source affected the Zn accumulation in the maize stems and leaves during the first growth stage followed by its remobilization from the stems to other plant organs during the second growth stage. The substantial increase in grain yield over control ensures the Zn_5_ (OH)_8_ (NO_3_)_2_·2H_2_O potential as a long-term foliar fertilizer. The result also proposed that the Zn transportation in maize organs strongly depends on the growth period. Initially, Zn is transported from the roots and leaves to the stem during the first growth period followed by its movement from the stem to other organs of the plant in the next stage [116]. Hence, detailed studies concerning the nanomaterials foliar application on plants including their uptake, translocation and transformation, behavior and phytotoxicity of these nanomaterials on plants are required. 

### 10.2. Macronutrients Nano-Fertilizers

Global demand for macronutrient-based fertilizers is estimated to increase to 263 Mt by 2050 [117]. The higher surface area, target delivery and penetrability of ENMs account for their efficiency over conventional fertilizers to meet the target. Additionally, excessive accumulation of macronutrients and their uncontrolled discharge to water bodies deteriorate the agro-ecosystems and water quality from conventional fertilizer sources. ENMs significantly reduce the overall application amount and thus account for their potential as alternative sources for these nutrients to minimize environmental impacts. The controlled release profile of nutrients through ENMs enable efficient delivery and thus enhances nutrient use efficacy by plants to improve growth and yield traits. Nano-enabled urea-modified hydroxyapatite (HA) released urea in a programmed manner while decreasing its solubility by incorporating it into a matrix of biocompatible HA NPs. The NPs provided a high surface to volume ratio for binding a large amount of urea molecules. ENMs enhanced the seed germination in rice relative to their bulk salts and control probably due to α-amylase activity and starch content. Further, the precursors used for HA i.e., Ca(OH)_2_ and H_3_PO_4_ indicated that the combination of Ca the P in the formulation with controlled-release probably contributed to plant growth over direct soil P application. Bentonite modified biochar have shown higher phosphate adsorption potential and superior P-slow release kinetics relative to unmodified biochar. The presence of Mg and Ca in bentonite led to the formation of Ca and Mg precipitations, porous structure and the reduced negative charge on the surface of the derived biochar. The tailored P-release kinetics of the prepared composite was achieved by varying the amount of bentonite [118]. Humic substances fabricated HA NPs were exploited for the interaction between the polyphenolic groups of humic substances and the surface of HA using *Zea mays* as a model crop over commercial fused superphosphate and bare HA NPs. The NCs positively affected early plant growth, corn productivity, resistance to salt stress and rhizosphere bacteria due to the synergistic co-release of phosphate ions and humic substances [119]. Foliar-applied CaO NPs to Ca-deficient peanut enhanced Ca accumulation with improved root development of the plants as compared to bulk CaO and CaNO_3_. The results confirmed the transportation of Ca through the phloem at the nanoscale; however, its mode of action is still unknown. Likewise, application of nano-CaCO_3_ in seed treatment of Vigna mungo improved plant growth particularly, shoot water content, as well as fresh and dry biomass relative to control and conventional bulk salts CaCl_2_ [120]. Integrated application of Mg ENMs (500 mg·L^−1^) and normal Fe (500 mg·L^−1^) to black-eyed pea (*Vigna unguiculata*) considerably increased seed mass (10%) over bare Fe application. However, the use of a relatively high concentration of sources limits their practical applicability for negative implications on non-target soil biotic components. Nitrogen and K doped CaP NPs in the form of amorphous (ACP) or nano-crystalline apatite (Ap) were synthesized. Amorphous CaP NPs are intrinsically rich in Ca and P; however, the concentration of NK dopants is small in comparison to the molar content of soluble conventional fertilizers. Doped NPs showed superior controlled release potential over the bare NPs. Moreover, comparable yields of wheat grains were obtained using nanoU-NPK, in much lower N quantity (a reduction of 40 wt%) over the conventional treatments [121]. 

## 11. Biofortification through Green Technology

Green technology includes the use of microorganisms for the improvement of the soil nutrient status by enhancing nutrient accessibility to the crops. Excessive use of chemical fertilizers possessed adverse effects on environmental ecology and human health. Thus, microbes containing biofertilizers may prove an eco-friendly efficient alternative to chemical fertilizers for plant growth and soil fertility. 

Plant growth-promoting microbes improve plant health by various direct and indirect mechanisms such as biological nitrogen fixation, the production of various plant growth hormones, hydrolytic enzymes, siderophores, HCN and solubilization of K, Zn and P [14]. Intrinsic plant-based strategies including secretions of chelators, organic acid production and phyto-siderophores are solely not sufficient to convert the nutrients in their bioavailable form. Chelating compounds produced by micro-organisms, form complexes with Zn and release them at the root surface which enhances the Zn availability and ultimately results in Zn biofortification in plants. Numerous studies are available on the beneficial role of microorganisms on the biofortification in crops. The major source of biofertilizers include bacteria and fungi (Table 6).

### 11.1. Bacterial Biofortification

The decline in soil pH with the application of *Pseudomonas* and *Bacillus* sp. converted soluble zinc complex compounds (ZnS, ZnO and ZnCO_3_) into zinc ions in broth culture. The qualitative and quantitative parameters related to Zn and Fe concentration of wheat crop were improved by the use of *Arthrobacter* sp. DS-179 and *Arthrobacter sulfonivorans* (DS-68) [122]. In another report, the inoculation of siderophore-producing *Arthrobacter sulfonivorans* DS-68 and *Enterococcus hirae* DS-163 endophytes in lesser Fe soil content resulted in an increased average number of root tips and root surface area by 1.6-fold and 2-fold, respectively, over the control. However, in case of high Fe content in soil, varied the average number of root tips and root surface area by 1.2-fold and 1.5-fold, respectively, over the control. These increased root parameters directly facilitated Fe fortification in wheat grains. Subsequently, the combined inoculation of *Bacillus subtilis* DS-178 and *Arthrobacter* sp. DS-179, promising Zn-solubilizer and siderophore-producing endophytes (*Enterococcus hirae* DS-163 and *Arthrobacter sulfonivorans* DS-68) resulted in improved yield and micronutrient status in wheat [106]. 

### 11.2. Endophytic Biofortification

The inoculation of mycorrhizal fungi positively influences the availability of micronutrients in soil owing to siderophore production and rhizospheric acidification besides hyphal transport of nutrients through the external mycelium. Further, the improved grain Zn concentrations were observed on the colonization of *Rhizophagus irregularis* under Zn-deficient conditions through upregulation of HvZIP13 [123]. The integrated use of PGPB and AM fungi reflected superior yield-related parameters with enhanced Zn and Fe grain concentration in wheat over control. The PGPB enhanced the various metabolic activities that contribute to plant growth parameters. However, AM participates in several mechanisms for efficient micronutrients uptake in plant tissues and stimulation of plant growth by the secretion of signaling molecules and various substances prompting modification in root morphology and anatomy of crop plants. Apart from that, combined use of PGPB and AM fungi confer better stress alleviation, weed control and protection against pathogens [124]. A quantity of earlier evidence is there that endophyte inoculation significantly decreased the phytic acid content in wheat grains by approximately 26% over the RDF with a consequent increase in Fe and Zn concentration [106].

## 12. Interactions between Foliar-Applied Minerals and Plant Nutrients

The nutritional status of crops and their application policies may pose synergistic or antagonist effects via leaf absorption, phloem mobility and accumulation of foliar-applied minerals in grains. Synergistic interaction between nutrients diminishes their deficiency by facilitating their uptake and translocation, whereas antagonistic interactions may intensify their deficiencies and toxicities of plant nutrients. Literature studies demonstrated that repeated foliar applications of a metal cation (Zn^2+^, Fe^2+^ and Cu^2+^), especially near the maturity stage, have negatively affected the mineral content of other metal cations (Table 7).

### 12.1. Micronutrients and Micronutrients

Owing to the competition between Fe and Zn, foliar Fe application adversely affects the grain Zn density and vice versa. The metal ions compete for the absorption by roots, loading into the xylem followed by chelation for translocation and cross membrane transport by particular carrier proteins. Furthermore, foliar-applied Cu suppressed the Mn content in grain [125]. The collective use of Fe/Zn and urea in foliar spray enhanced the grain Zn and Fe concentration in chickpea over bare Fe/Zn treatment. The observed trend attributed to the higher root uptake and remobilization of micronutrients in the plant due to elevated Fe and Zn transporter protein content by urea that improved the various plant function. In another report, urea promoted the translocation of Fe by the combined use of urea + ^59^Fe-EDTA, owing to its contribution in the Fe chelation through nitrogen compounds and transportation of the leaf—Fe was absorbed to different organs [127]. A negative correlation between Fe-Mn and Fe-Cu has been reported on Fe fertilization due to antagonism and the same transporter lines in teosinte [61]. Although incorporating various minerals in a single application may overcome these negative interactions, the fusion of multiple minerals requires more salt contents in spray solution, which ultimately leads to the intense risk of leaf burning. Thus, while adapting foliar intervention the optimum concentration of the nutrient should be applied. 

In the case of Se and I, the source used for foliar feeding determines the interaction between different nutrients. Foliar-applied Se (in the form of Na_2_SeO_4_) revealed synergistic interaction with Fe and enhanced its concentration in wheat crop over control [126]. The trend attributed to the similar effect of NO_3_^−^ and selenate (SeO_4_^2−^) on uptake and mobility of Fe and Zn. Interestingly, in case of iodate (IO_3_^−^) and NO_3_^−^, the competitive mobilization exists due to a similar transportation mechanism from leaves to the grains [6]. Furthermore, IO_3_^−^ showed positive interactions with P in contrast to iodide (I^−^) that decreased the P content in grains [128]. However, the exact mechanism behind this variable response is not yet known.

### 12.2. Micronutrients and Macronutrients

Literature studies demonstrated the variation in macronutrient concentration on micronutrient application in crops. Nitrogen showed a synergistic effect with multiple mineral (Fe, Zn and Cu) accumulation in grains as well as on shoot remobilization via soil and foliar application in various cereal crops. Literature studies demonstrated the significant increase in grain Zn content with soil fertilization and foliar feeding of N, both with and without foliar Zn application [129]. Similarly, soil and foliar application of N enhanced the grain concentration of soil and foliar-applied Fe. The foliar application of Fe (as Fe-EDTA) in durum wheat resulted in facilitated N uptake and grain accumulation [130]. Further, NO^3−^ (applied as KNO_3_) facilitates the mobility of I in the phloem along with the role of KNO_3_ as a surfactant in the spray solution for the penetration of I from the surface of the leaf [6]. Surprisingly, foliar feeding of minerals showed a relation with N root uptake capacity and grain concentration. Foliar-applied Zn and Fe in the form of ZnSO_4_ and FeSO_4_, respectively, enriches the grain with N content and crude proteins. Nutrient cocktail application including N, P and Zn improved dry matter, grain yield and uptake of N, P, K, and Zn in rice over sole N application accounts for the importance of complete and balanced fertilization. The results confirmed the synergistic effect of P and K application along with N and the positive interaction of N and K. 

In comparison to N and P, the interaction of foliar application of K and plant nutrient status has been rarely studied in food crops. Foliar feeding of K may result in the grains enriched with Zn and Fe due to remobilization of nutrient reserve into grains. For example, optimum supply of K encouraged the mycorrhizal association that lead to enhanced Zn uptake even in the presence of P application [131]. Foliar-applied Zn + K significantly enhanced Zn content in wheat over bare Zn application [129]. 

In contrast to N, P has shown antagonistic interactions with the biofortification of other nutrients. The results attributed to the presence of most of the P (60–80% of total plant and 90% of total grain P) in the form of phytic acid in cereals. Phytic acid forms insoluble complexes with metals, thus acting as an antinutrient factor by decreasing their bioavailability for digestion in the human body. Application of P ultimately enhanced the phytate contents in cereal grains thus decreasing the uptake and grain accumulation of metal nutrients. Alternatively, foliar-applied Zn and Fe significantly reduced phytate content in cereal grains [132]. Numerous reports showed that soil and foliar application of P induce the Zn deficiency owing to the antagonistic interaction between P and Zn. Wang et al. showed that foliar-applied mixture (Zn + P) declined grain Zn concentration by 26% in comparison to bare Zn application [133]. The trend attributed to the formation of Zn-phosphate on the surface of the leaf, thus impeding the Zn diffusion into leaves. The other different mechanisms that account for the antagonistic P-Zn interactions involved the (i) suppression of mycorrhizal root activity that causes the Zn uptake from soil to root, and (ii) the elevation of P concentrations encouraging Zn-binding cell wall properties that immobilize Zn in shoots/roots, thus diminishing its translocation [131]. 

### 12.3. Micronutrient and Heavy Metals

Rapid urbanization, application of fertilizers and sewage sludge as well as mining and smelting led to heavy metal (HM) accumulation in excess amounts in paddy soils. Ingestion of HM by humans can severely affect the skeletal, nervous, circulatory, endocrine, enzymatic and immune systems. Excessive accumulation of Zn in soil synergistically affects the crop uptake of other HMs and further results in toxicity in humans and livestock. Hussain et al. reported that Zn-biofortified wheat cultivar Zincol-2016 was more resistant to soil Cd contamination for straw and grain yield than standard wheat cultivar (Faisalabad) [134]. Further, Zn fertilization to Cd-spiked pots through soil + foliar application enhanced grain yield and grain Zn concentration with a simultaneous decrease in grain Cd concentration over sole soil application. However, both cultivars, when grown in Cd spiked soil, contained grain Cd content above the permissible limit (0.20 mg kg^−1^), except standard cultivar treated with soil + foliar Zn. Relatively lower grain Cd concentration was observed in standard cultivar over Zn-biofortified cultivar. Higher effectiveness of foliar Zn application on Zincol-2016 over standard cultivar reflected the complementary effect of agronomic and genetic biofortification. Nitrogen fertilization enhanced Cd and Zn uptakes while inhibiting their translocations in dwarf polish wheat. Under a lack of NH_4_^+^, Cd significantly reduced Zn uptake and promoted Zn translocation, while the supply of NH_4_^+^ Cd slightly reduced the Zn uptake, thus not affecting its translocation. Thus, NH_4_^+^ treatment alleviated Cd-induced inhibition of Zn uptake and partly reduced the promotion of Zn translocation. The different phenomena such as changes in N content, chemical forms of Cd, and the subcellular distribution of Zn and Cd might have influenced the uptake of Zn and Cd and translocation from root to shoot. Thus, NH_4_^+^ application may significantly affect Cd/Zn interactions in different field crops and thus the accumulation of toxic metals. The negative impact of Cd contamination due to oxidative stress on the plant growth, grain yield and grain Zn concentration has been reported in bread wheat, which could be overcome by a high intrinsic seed Zn concentration (49 mg kg^–1^), due to reduced lipid peroxidation and improved stand establishment. Further, the combined application of Zn + biochar reduces grain Cd concentration with enhanced grain Zn concentration over sole applications of Zn and biochar [78]. Engineered ZnO NPs positively affects growth, photosynthesis, grain yield and grain Zn concentration, while decreasing Cd concentrations in wheat over control. The ZnO NPs increased SOD and POD activities in leaves of wheat and inhibited the electrolyte leakage, which could thus significantly reduce the toxicity and increase Zn concentration in cereals to reduce hidden hunger. Dual-purpose Se and Si NPs were explored for Se biofortification of brown rice with a simultaneous decrease in Cd and Pb concentration. The combined foliar application of both NPs significantly reduced the Cd and Pb contents over control and sole applications by decreasing oxidative stress and increasing Se content. Foliar NPs application also enhanced protein content and decreased phytic acid content [132]. Integrated biochar and zerovalent iron application reduced Cd accumulation by 83% due to facilitated sorption, which resulted in enhanced Zn and Fe concentration in rice. Rhizopheric micro-organism *R. sphaeroides* exhibited phytoremediation potential for Cd and Zn contaminants in the soil through varied Cd and Zn chemical fractions. *R. sphaeroides* reduced the bioavailable soil fractions of Cd and Zn, and reduced Zn and Cd accumulation in wheat, yet it enhanced the Zn/Cd ratio in wheat, thus they are comparable to the chemical immobilization approach for remediation of heavy metals. Foliar-applied Zn-lys significantly enhanced enzyme activities, photosynthesis, grain yield and grain Zn contents in wheat. The chelate source also reduced Cd contents in grains, root and shoot as well as oxidative stress linearly in a dose-additive manner. Zn presumably inhibited the oxidative stress in plants under heavy metal stress and amino acid decreased the ROS production in plants. Thus, Zn-lys enhanced the antioxidant enzyme activities over control probably due to lower Cd in plants which enhance plant growth due to the plant tolerance to Cd stress. Foliar application of Zn (3 g L^−1^ ZnSO_4_) at booting stage enhanced shoot and grain mineral concentration including P, K, Na and Zn with decreases in grain Cd concentration due to sufficient Zn supply in the shoot (particularly in flag leaf) of wheat crop. However, soil-applied Zn only improved the grain mineral content and plant growth under Cd stress but did not affect the grain Cd concentration of wheat. A decrease in root Cd content while increasing flag leaf concentration in soil application demonstrated the increased translocation from root to shoot [135]. Thus, the results account for the significance of foliar application to reduce heavy metal accumulation in cereals over soil application.

Foliar-applied Se and Zn decrease Cd accumulation in wheat grown under Cd stress while promoting plant growth, enzymatic antioxidant defense system and photosynthetic [109]. Integrated use of fly ash and horse manure enhanced grain yield and grain Se concentration as well as effectively reduced Cd accumulation in rice grain. Fly ash reduced the soil-Cd availability and thus the translocation of Cd within the plant, due to increased Si content. Horse manure increased the amount of root Fe plaque, which prevented Cd from entering root cells and thus reduced the Cd accumulation in the grain. Thus, the combined use of FA and HM is an effective amendment treatment to tackle Cd stress with improved plant growth and Se concentration [136]. The overall interactions between micronutrients and heavy metals have been reported in Table 8.

## 13. Crop Yield Responses to Mineral Applications

### 13.1. Soil Application

Soil application of micronutrients has shown tremendous effects on crop yield responses (Table 9). 

Dhaliwal et al. reported a significant increase in grain yield of wheat at research farm and farmer’s field up to 12.05% and 28.6% respectively, with soil Zn application (ZnSO_4_·7H_2_O @ 62.5 kg ha^−1^) [50]. Ram et al. revealed that the soil Zn application increased grain yield of wheat and rice up to 50% and 14.8%, respectively [51]. Site-specific Zn application response to different lentils was evaluated by Maqsood et al. [137]. Zinc application resulted in a significant increase in grain yield along with grain Zn application over the control on some low organic matter, high pH Brown Chernozem soils. Up to 40% in grain yield was observed for small red lentils with 5 kg ha^−1^ Zn application. Similar results were obtained by Yaseen and Hussain [138], where Zn application (12 mg kg^−1^) to biofortified wheat cultivars Zincol-2016, rather than Jauhar-2016, resulted in improved grain yield with a simultaneous decrease in Fe and phytate concentration over control. ZnSO_4_ fertilization at the rate 10 mg kg^−1^ considerably increased (44.7%) gain yield in mung bean, whereas a 25% increment was observed in bread wheat with 20 kg ha^−1^ [139]. Soil Zn application to oats fodders resulted in a 28.9% increase in forage yield [63].

### 13.2. Foliar Application

Indeed, foliar application of minerals enhances grain yields, particularly applied at the appropriate time and rate (Table 10). For maximum yield response, micronutrients are susceptible to soil application at the time of sowing. If the nutrient availability is limited from the soil, they are sprayed through foliar application during the specific vegetative or fruiting stages, such as early jointing and late tillering. 

This early application ensures that nutrients are supplied at the time of high demand of crop. The late application during the reproductive stage enhances grain mineral content but may not enhance grain yield of crop. The foliar application also facilitates rapid correction of severe deficiencies during the early stages of growth. In different maize cultivars, foliar-applied Fe (1%) at different stages of plant growth (knee-high, pre-tasseling and post-tasseling) improved the grain yield up to 6.4% [59]. Foliar Fe application with three sprays (2% FeSO_4_ after 30, 37 and 44 days of sowing) improved the herbage yield of teosinte up to 32.53% [61]. In forage crops, foliar-applied Cu enhanced the improved the oats fodder yield up to 28.9% [47]. On the other hand, the maximum increase in oats yield was found to be 24.6% with foliar Zn application [63]. Ramzan et al. observed the improved yield traits on foliar application of bare and combination of Zn and Fe (0.5% ZnSO_4_ + 1% FeSO_4_) over soil fertilization in wheat crop [141]. Foliar fertilization using bio-stimulant enhanced the micronutrient content and grain yield in maize crop due to the chelating effect of micronutrients that facilitate the absorption and transportation of micronutrients inside the plants. Se and I are essential elements for humans and animals, but not for plants. Foliar fertilization of Se and I enhanced their concentration in cereal grains [6]. Surprisingly, foliar-applied Se (as SeO_4_^2−^) enhanced the yield of wheat, while maintaining turgor and gas exchange characteristics, thus confirming the beneficial role of SeO_4_^2−^ in plants. Foliar-applied Mn-EDTA (243.80 in 2006 and 257.74 kg ha^−1^ in 2007) showed significant results from yield perspectives in beans over soil application (166.97 in 2006 and 180.60 kg ha^−1^ in 2007) [140]. 

Further, the yield response through the foliage is concentration-dependent, as excess concentrated foliar-solutions may burn leaves accounting for yield losses [142]. The studies proposed that the relative increase in yield with the foliar feeding was lower than soil application, owing to the substantial yield losses due to low nutrient availability during the active vegetative growth [50]. Numerous factors including additional labor cost, variable yield response due to leaf burning and the need for costly instruments restrict the implementation of the intervention. In target countries, government policies should be implemented to encourage mineral fertilization, such as subsidies and quality price of high mineral grains. Although such decisions will intensify the government expenditure on agriculture, certainly decrease the budget on health and increase average family income with potential health benefits.

## 14. Conclusions and Future Perspectives

In this article, we reviewed the strategies and applications of biofortification of various field crops for nutritional security. Globally, Zn deficient soils are more prevalent than other micronutrient deficiencies. The essentiality of micronutrients has been recognized for humans and livestock, where micronutrient deficiency may lead to adverse health consequences. The high reliance of human population on cereal-based intakes is the primary cause for micronutrient deficiency in developing and underdeveloped countries. Agronomic biofortification through fertilization seems sustainable and cost-effective to overcome micronutrient deficiency in the human population. Management practices such as organic or inorganic inputs may either act as a direct nutrient source or may contribute to enhancing micronutrients bio-availability through alteration in soil properties. However, due to excessive use of fertilizers, there is a dire need for a complementary approach. Transgenic biofortification is a versatile approach to improve micronutrient content in crops; however, less work is conducted in the area of fodder crops. Green technology includes the use of microbes due to their vital role in improving nutrient availability and uptake, further studies are required concerning the selection of suitable microbial culture. Enrichment of plant nutrients through nano-fertilizers is an emerging area of research, hence demanding detailed studies associated with the toxicity issue. Thus, we contest that the information provided here will aid in addressing future nutritional security and thereby alleviate the prevalence of micronutrient deficiencies. 

## Figures and Tables

**Table 1:** Soil application of micronutrients for mineral fertilization.

Micronutrient	Crop	Reference
Zn	Wheat	[50]
Zn	Rice, Wheat	[51]
Zn	Cowpea	[52]
Zn	Lentil	[53]
Zn + Se	Soybean	[54]

**Table 3:** Seed treatment with micronutrients for mineral fertilization.

Seed treatment	Micronutrient	Crop	Reference
Seed Priming	B	Wheat and Rice	[66]
	Zn + *Pseudomonas* sp. MN12	Bread-wheat	[67]
	Fe and Zn	Wheat and Barley	[68]
Seed Coating	Zn	Wheat	[69]
	B	Rice	[70]
	Mn	Bread wheat	[60]

**Table 5:** Micronutrient nano-fertilizers for biofortification.

Micro-Nutrient Nano-Fertilizer	Crop	Reference
ZnO NPs	Wheat	[113]
Fe_3_O_4_	Wheat	[114]
Graphene oxide-Zn NPs	Wheat	[115]
Graphene oxide-Cu NPs	Wheat	
Zn_5_(OH)_8_(NO_3_)_2_·2H_2_O	Maize	[116]

**Table 6:** Biofortification through green technology.

Microorganisms	Micronutrient Affected	Crop	Reference
*Arthrobacter* sp. DS-179 and *Arthrobacter sulfonivorans* (DS-68)	Fe and Zn	Wheat	[122]
*Bacillus subtilis* DS-178 and *Arthrobacter* sp. DS-179, *Enterococcus hirae* DS-163 and *Arthrobacter sulfonivorans* DS-68	Fe and Zn	Wheat	[106]
*Rhizophagus irregularis*	Zn		[123]
PGPB and AM fungi	Zn and Fe	Wheat	[124]
Endophytes	Fe and Zn	Wheat	[106]

**Table 7:** Interaction between micronutrients and trace elements.

Micronutrient	Micronutrient and Trace Element	Type of Interaction	Crop	Reference
Cu	Mn	Antagonistic		[125]
Fe	Se	Synergistic	Wheat and rice	[126]

**Table 8:** Interaction between micronutrients and heavy metals.

Micro-Nutrient Involved in Interaction	Heavy-Metal Stress	Mode of Application	Crops	Reference
Zn	Cd	Soil + foliar	Wheat	[134]
Zn	Cd	Seed treatment	Wheat	[78]
Zn + Biochar	Cd		Wheat	
Se and Si	Cd and Pb	Foliar	Brown rice	[132]
Zerovalent iron + Biochar	Cd		Rice	[79]
Zn	Cd	Foliar	Wheat	[135]
Se + Zn	Cd	Foliar	Wheat	[109]

**Table 9:** Crop yield responses to soil mineral application.

Micronutrient	Rate	Crop	Increase in Yield (%)	Reference
Zn	62.5 kg ha^−1^	Wheat	28.6	[50]
Zn	50.0 kg ha^−1^	Wheat	50.0	[51]
Zn	50.0 kg ha^−1^	Rice	14.8	
Zn	5.0 kg ha^−1^	Lentils	40.0	[137]
Zn	12 mg kg^−1^	Wheat (Zincol-2016)	22.6	[138]
Zn	10 mg kg^−1^	Mung-bean	44.7	[139]
Zn	25 kg ha^−1^	Oats	28.9	[63]

**Table 10:** Crop yield responses to foliar mineral application.

Micronutrient Applied	Crop	Increase in Yield (%)	Reference
Fe	Maize	6.4	[59]
Zn	Maize	6.2	
Fe	Teosinte	32.5	[61]
Urea	Chickpea	12.4	
Urea	Chickpea	10.5	
Cu	Oats	28.9	[47]
Zn	Oats	24.6	[63]
Zn + Fe	Wheat	24.8	[140]

## Data Availability

Not applicable.
